# Trends and variability of the atmosphere–ocean turbulent heat flux in the extratropical Southern Hemisphere

**DOI:** 10.1038/srep14900

**Published:** 2015-10-09

**Authors:** Agnieszka Herman

**Affiliations:** 1 Institute of Oceanography, University of Gdansk, Poland

## Abstract

Ocean–atmosphere interactions are complex and extend over a wide range of temporal and spatial scales. Among the key components of these interactions is the ocean–atmosphere (latent and sensible) turbulent heat flux (THF). Here, based on daily optimally-interpolated data from the extratropical Southern Hemisphere (south of 30°S) from a period 1985–2013, we analyze short-term variability and trends in THF and variables influencing it. It is shown that, in spite of climate-change-related positive trends in surface wind speeds over large parts of the Southern Ocean, the range of the THF variability has been decreasing due to decreasing air–water temperature and humidity differences. Occurrence frequency of very large heat flux events decreased accordingly. Remarkably, spectral analysis of the THF data reveals, in certain regions, robust periodicity at frequencies 0.03–0.04 day^−1^, corresponding exactly to frequencies of the baroclinic annular mode (BAM). Finally, it is shown that the THF is correlated with the position of the major fronts in sections of the Antarctic Circumpolar Current where the fronts are not constrained by the bottom topography and can adjust their position to the atmospheric and oceanic forcing, suggesting differential response of various sections of the Southern Ocean to the changing atmospheric forcing.

Many aspects of the ocean–atmosphere interactions, especially in the remote, sparsely monitored extratropical Southern Hemisphere (SH), are poorly understood^1^. This is related to, among other things, our limited knowledge concerning patterns of temporal and spatial variability of relevant quantities, including the two components of the surface latent and sensible turbulent heat flux (THF). Although, globally, the largest THF values occur in vicinity of major oceanic frontal zones (like the Gulf Stream, Kuroshio or Agulhas Current), the contribution of THF to the local ocean–atmosphere net heat exchange is substantial over most oceanic regions^1^,^2^,^3^,^4^. Contrary to the longwave and shortwave radiative energy flux, which is determined by weather patterns spanning the whole troposphere, the THF directly depends on processes taking place within the atmospheric boundary layer (ABL) and the surface ocean mixed layer (OML). However, the THF is a building block of a number of feedbacks through which it indirectly affects/is affected by important components of the climate system, extending far beyond the ABL/OML. In particular, anomalies of the surface heat flux associated with the Southern Annular Mode (SAM), one of the dominant modes of the Southern Hemisphere atmospheric variability, drive changes in the oceanic mixed-layer depth (MLD) in the Southern Ocean^5^. In general, air–water heat flux dominates the OML heat budget and its seasonal variations in all regions of the Southern Ocean^6^,^7^, with significant contributions from geostrophic and Ekman advection in vicinity of major fronts of the Antarctic Circumpolar Current (ACC). Results of modeling studies suggest that localized sources of surface heat flux, e.g., associated with oceanic fronts, may play an important role in maintenance of atmospheric baroclinicity and the storm track formation^8^,^9^,^10^. Within the ACC, where water masses with a very wide range of properties are in contact with the atmosphere, the THF contributes to modification of these properties and thus, indirectly, to water-mass formation and the overturning circulation in the ocean^11^. THF variability influences also the SAM-related sea surface temperature (SST) anomalies^4^ and presumably is partly responsible for the observed trends of the SH upper-ocean heat content^12^. For the role of THF in frontal zones, see^1^,^13^,^14^ and references there. THF also plays a dominant role in extreme ocean–atmosphere heat flux events, contrary to the radiative fluxes that have a much longer scale of variability and dominate the seasonal cycle^15^.

Although a number of datasets—including the one used in this study—provide daily fields of the THF components, their analysis typically concentrates on seasonal and/or interannual time scales and large, hemispheric spatial patterns^16^,^17^,^18^,^19^. The main purpose of this work is to contribute to this broad picture of the turbulent ocean–atmosphere heat exchange by including the short-term, day-to-day and synoptic THF variability. At many locations in the Southern Ocean (see Supplementary Figure 1 for an example), this short-term THF variability clearly dominates over longer, seasonal signal. Our analysis concentrates on the extratropical ocean in the Southern Hemisphere (south of 30°S; see Fig. 1) and covers the period 1985–2013. The aim is to identify areas with statistically significant changes in the properties (median, standard deviation and skewness) of probability distribution functions (pdfs) of the latent and sensible heat flux, to relate them to changes in the meteorological and hydrographic variables influencing turbulent heat exchange between the atmosphere and the ocean, and to interpret the observed trends in the context of the large-scale southern hemisphere climate-change patterns observed in the last decades. Particular emphasis will be given to the existence of quasi-periodicity in the THF and variables influencing it. We also discuss the influence of the changing position of the ACC fronts on the THF variability.

This study is based on the data from the Woods Hole Oceanographic Institution Objectively Analyzed air-sea Fluxes (OAFlux) database, created from a number of combined satellite-derived and model-based data sources. As described in Methods and in the Supplementary Note S1, the OAFlux data cover a sufficiently long time period to facilitate the trend analysis presented in this work and, contrary to purely-satellite products, don’t contain gaps (which is important for spectral analysis). Most importantly, the data have been successfully validated against the only available source of systematic observational data from the domain of study, with considerably higher correlation coefficients and lower standard deviation of differences than obtained for another, reanalysis product (see Supplementary Note S1.2 for details).

The OAFlux data set contains global fields of the surface meteorological variables necessary for the calculation of the latent and sensible heat flux at the ocean–atmosphere interface: the sea surface temperature *T*_*s*_, the air temperature *T*_*a*_ and specific humidity *q*_*a*_ at 2-m height, and the wind speed *U*_10_ at 10-m height. The final global daily fields of *T*_*s*_, *T*_*a*_, *q*_*a*_ and *U*_10_, together with their error estimates, are compiled from various sources, including a number of satellite and reanalysis products^16^,^17^. The latent and sensible heat fluxes, *F*_*lh*_ and *F*_*sh*_, respectively, are calculated from the following bulk formulae:


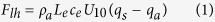


and


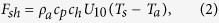


where *ρ*_*a*_ denotes the air density, *L*_*e*_—the latent heat of evaporation of water, *c*_*p*_—the specific heat of water, and *c*_*e*_ and *c*_*h*_ are turbulent exchange coefficients. The near-surface specific humidity 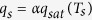, where *q*_*sat*_ denotes the saturation humidity at *T*_*s*_, and the coefficient *α* = 0.98 accounts for the salinity-induced reduction of *q*_*sat*_^17^. As follows from (1) and (2), positive *F*_*lh*_ and *F*_*sh*_ values mean heat flux from the ocean to the atmosphere. Divided by *ρ*_*w*_*L*_*e*_ (where *ρ*_*w*_ denotes water density), *F*_*lh*_ is a measure of the rate of evaporation from the sea surface. The daily OAFlux data, used in this study, are available on a 1-degree resolution grid in the 29-year period 1985–2013. Additionally, to put the THF analysis in a broader context, the net longwave and shortwave radiation data, *F*_*lw*_ and *F*_*sw*_, from the International Satellite Cloud Climatology Project (ISCCP) are used at selected stages of the analysis (see Methods). They are available within a shorter period 1985–2009, and are interpolated onto the OAFlux grid from the original 2.5-degree resolution. For comments on the quality of the OAFlux data in the Southern Ocean, see Methods and Supplementary Note S1.

## Spatial and temporal variability of THF

Not surprisingly, the broad spatial patterns of the daily *F*_*lh*_ and *F*_*sh*_ agree with their long-term variability obtained from OAFlux and other similar datasets^16^,^17^,^18^,^19^,^20^. As shown in Fig. 2, the latent heat flux tends to increase equatorwards, with median values close to 100 W/m^2^ along the northern edge of the study domain. The largest values occur along the western and eastern coasts of Australia, within the Agulhas Current/Agulhas Retroflection, and in the Atlantic Ocean east of South America, within the Brazil Current close to the edge of the southbound branch of the Subantarctic Front (SAF) loop (Fig. 2a,b). The median values of *F*_*sh*_ are generally smaller than those of *F*_*lh*_. Although in areas with largest *F*_*lh*_ the sensible heat flux is large as well, there are a number of other local *F*_*sh*_ maxima over various sections of the Southern Ocean (Fig. 2d,e), where both heat flux components have comparable magnitude. Not surprisingly, areas with negative median sensible heat flux, i.e., *T*_*a*_ > *T*_*s*_, coincide with areas of the lowest (close to zero) latent heat flux. Contact of relatively warm air with a cold sea surface stabilizes the lowest atmosphere and prevents moisture from being mixed upward, thus leading to an increase of *q*_*a*_ in the lowest atmosphere, to small (*q*_*s*_ − *q*_*a*_) differences, and thus to limited evaporation. Another typical situations with large *F*_*sh*_ and small *F*_*lh*_ occur when both air and water temperature are very low. Because, obviously, 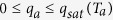 and 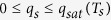, the difference (*q*_*s*_ − *q*_*a*_) lies within the range 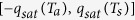. As the saturation humidity decreases very fast with temperature, it constrains possible latent heat flux values in cold conditions. Consequently, south of the ACC fronts *F*_*lh*_ rarely exceeds 50 W/m^2^ with a standard deviation of typically 20–30 W/m^2^ (Figs 2a,b and 3a,b). The temperature difference (*T*_*s*_ − *T*_*a*_) in such conditions tends to be positive, because *T*_*s*_ is limited from below by the freezing temperature of water. Close to the Antarctic continent, *F*_*sh*_ may dominate over *F*_*lh*_, especially when very cold air is advected from the south—the values of the standard deviation of *F*_*sh*_ along the southern boundary of the analyzed domain reach 40–50 W/m^2^ and are comparable to those in vicinity of major SST fronts, for example the Agulhas Return Current (Fig. 3d,e). The constrains described above are also responsible for the asymmetry of the pdfs of the turbulent fluxes in that region, with the skewness of 1.5–2 and 3–4 for the pdfs of *F*_*lh*_ and *F*_*sh*_, respectively (Fig. 4). However, over large areas of the Southern Ocean far from the Antarctic coast and the sea-ice edge, median *F*_*sh*_ is close to zero or even negative, i.e., in average conditions the heat is transferred from the atmosphere to the underlying ocean^11^, and the pdfs become narrow and symmetrical, especially within the Indian Ocean sector of the ACC, where the skewness of the pdfs of both *F*_*lh*_ and *F*_*sh*_ is close to zero (Fig. 4).

## Heat flux trends

Although the large-scale, dominant spatial patterns described above did not change within the analysis period, the pdfs of *F*_*lh*_ and *F*_*sh*_ in the decades 1985–1994 and 2004–2013 are different over large parts of the study domain (panels c, f in Figs 2, 3, 4). At the majority of locations, the differences between the two outermost decades accumulated gradually throughout the analysis period, i.e., the moments of the pdfs exhibit consistent trends throughout the whole analysis period (see Methods, Supplementary Fig. 6, and^16^,^18^ for analogous trends in annually averaged variables).

**Figure 1:**
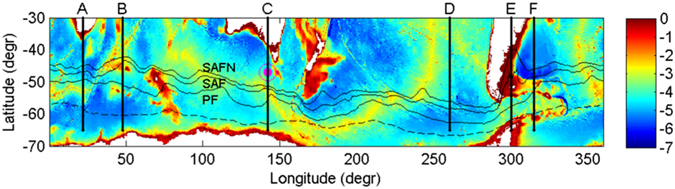
Ocean bottom topography (depths in km) of the extratropical Southern Hemisphere. The southern boundary of the area of study (defined as locations remaining ice-free during at least 70% of the time) is marked with a dashed line. The magenta point to the SW of Tasmania shows the location of the SOFS buoy (see Methods). The locations of the profiles (**A**–**F**), discussed in the text, are shown with thick continuous lines. Thin black contours mark the average position of the major ACC fronts (PF—Polar Front, SAF—Subantarctic Front, SAFN—nortern branch of SAF), based on AVISO data from the period 1992–2013 30.

**Figure 2:**
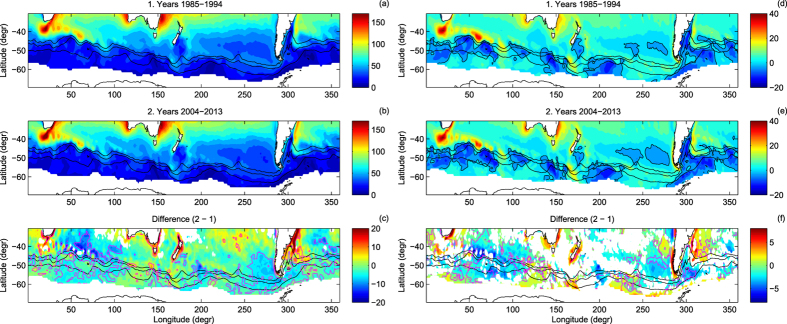
Median daily ocean-atmosphere latent heat flux *F*_*lh*_ (**a**–**c**) and sensible heat flux *F*_*sh*_ (**d**–**f**), in W/m^2^, in the area of study in the first (**a**,**d**) and last (**b**,**e**) decade of the analyzed period, and the change between them (**c**,**f**). In (**c**,**f**), areas with statistically insignificant differences between the respective pdfs (at a 99% confidence level) are left blank; magenta contours mark regions where the relative change was larger than ±10%. Thick black contours in (**d**,**e**) have value of zero. Note different color scales in the left and right panels.

Remarkably, although the median values only locally changed by more than 10% (magenta contours in Fig. 2c,f), changes regarding the width and shape of the pdfs are more substantial. Apart from the maps, they are illustrated along selected meridional profiles in Fig. 5 and Supplementary Figs 7–11 (see Fig. 1 for the profiles’ location), as well as in terms of the joint *U*_10_ − (*T*_*s*_ −*T*_*a*_) and *U*_10_ −(*q*_*s*_ −*q*_*a*_) pdfs at selected points along profiles B and F in Fig. 6 and Supplementary Figs 12–15.

**Figure 3:**
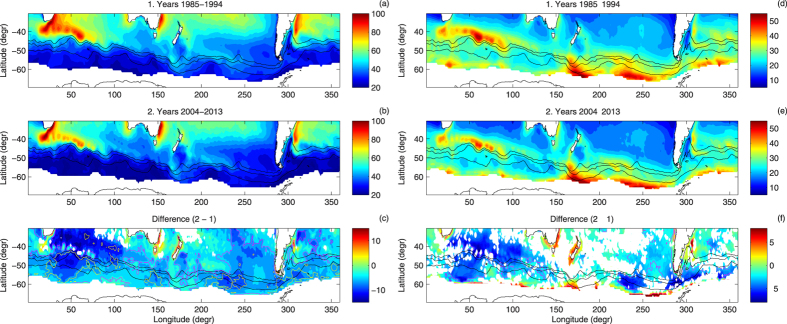
As in Fig. 2, but for the standard deviation of *F*_*lh*_ and *F*_*sh*_ (in W/m^2^). Yellow contours in (c) mark regions where the relative change was larger than ±20%.

**Figure 4:**
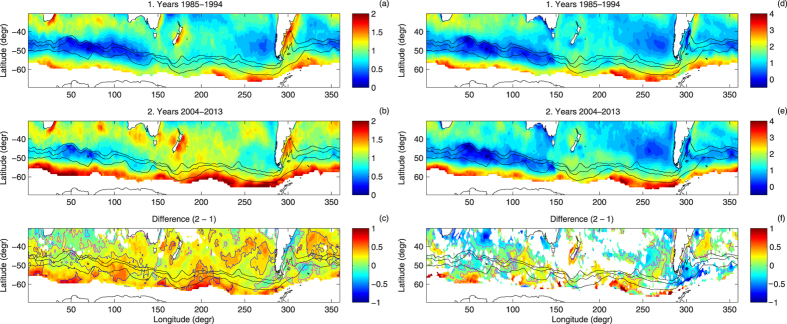
As in Fig. 2, but for the skewness (nondimensional) of the pdfs of *F*_*lh*_ and *F*_*sh*_. Blue contours in (c) mark regions where the relative change was larger than 50%.

**Figure 5:**
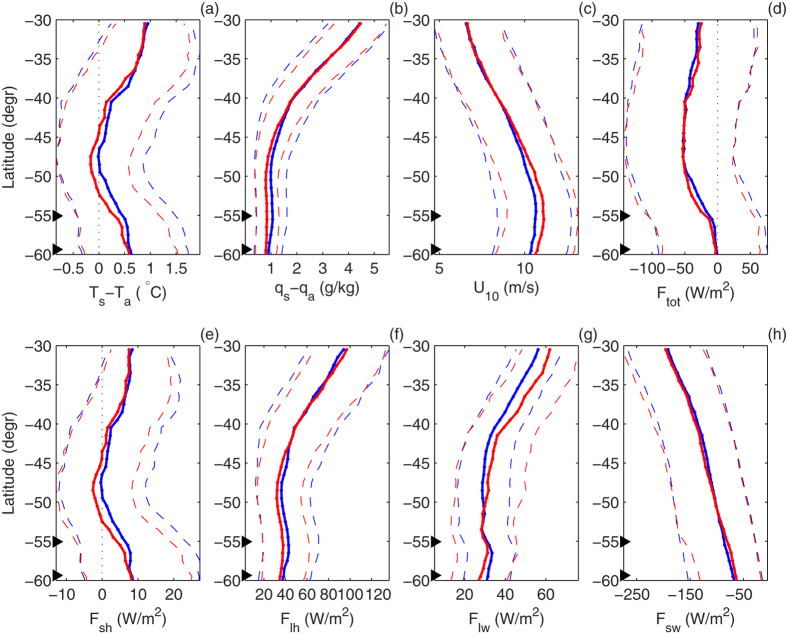
Changes of the analyzed variables ((**a**): temperature difference *T*_*s*_ − *T*_*a*_; (**b**): humidity difference *q*_*s*_ −*q*_*a*_; (**c**): wind speed *U*_10_; (**d**): total heat flux *F*_*tot*_; (**e–h**): sensible *F*_*sh*_, latent *F*_*lh*_, longwave *F*_*lw*_ and shortwave *F*_*sh*_ heat flux, respectively) along a meridional profile at 100°W (line D in Fig. 1): median values (thick continuous lines), 0.25 and 0.75 quartiles (thin dashed lines) in the first (1985–1994; blue) and last (2004–2013; red) decade of the analyzed period. Black triangles mark the average positions of the SAF and SAFN. Note that 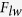, 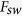 and 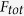 in the second time period are limited to 2003–2009. See Supplementary Figures 7–11 for profiles A–C, E and F.

**Figure 6:**
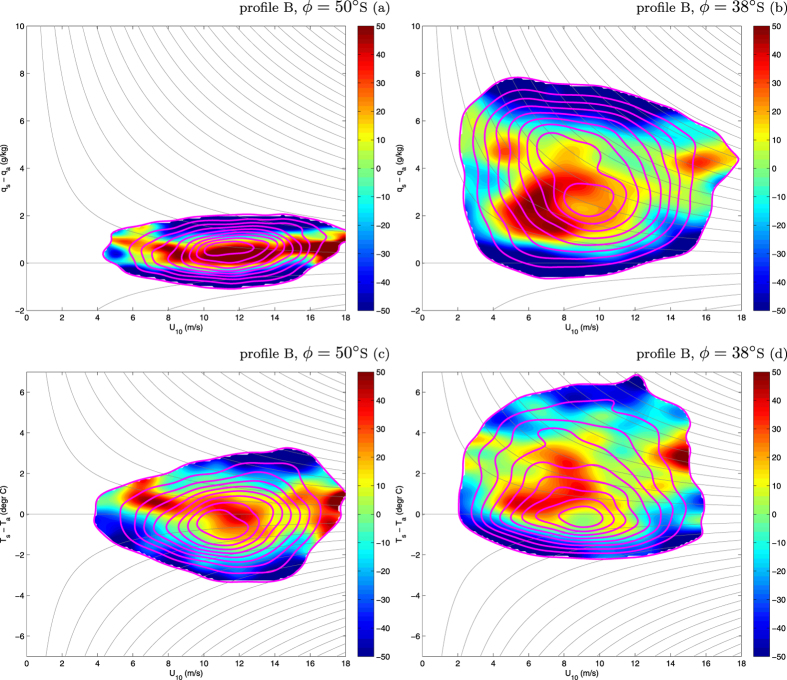
Joint pdfs of (*U*_10_, *q*_*s*_ − *q*_*a*_) and (*U*_10_, *T*_*s*_ − *T*_*a*_) in selected points along the profile at 48°E (line B in Fig. 1): 50°S (a,c) and 38°S (b,d). Magenta lines show the pdfs in the 1985–1994 decade, with contours drawn from 0.1 to 0.9, every 0.1 (normalized values). The colors show the relative change (in %) of the pdfs during the analysis period. Thin gray lines show contours of constant latent heat flux (**a**,**b**; drawn every 30 W/m^2^) and sensible heat flux (**c**,**d**; drawn every 10 W/m^2^). See Supplementary Figs 12, 13 for more points along profile B, and Supplementary Figs 14, 15 for profile F.

As noted in a number of observational and modeling studies, the last decades witnessed a positive trend in zonal wind speed over the Southern Ocean, combined with a southward shift in the location of the zone of the highest wind speeds (i.e., prevalence of the positive phase of the SAM index^21^,^22^). This trend is present in the analyzed dataset as well (panel c in Fig. 5 and Supplementary Figs 7–11). However, its influence on the turbulent heat flux components remains rather limited—both in terms of the median and the width of their pdfs. Only a significant increase in the skewness (Fig. 4), in the case of *F*_*lh*_ in some regions exceeding 50%, can be interpreted as a consequence of strengthening winds, especially if one considers that the increase of the mean wind speed is typically associated with higher wind speed variance and more frequent occurrence of very strong wind events^23^. The observed—and over large areas (especially south of ~40°S) negative—change in the variance of *F*_*lh*_ and *F*_*sh*_ (Figs 3 and 5 and Supplementary Figs 7–11) can be mostly attributed to changes in the (*T*_*s*_ −*T*_*a*_) and (*q*_*s*_ −*q*_*a*_) differences, which decreased at the majority of the analyzed locations (Figs 5 and 6, Supplementary Figs 7–15). Within the ACC, the interquartile range of *F*_*sh*_ and, especially, *F*_*lh*_ decreased. The joint pdfs show that the decrease in probability of large ocean–atmosphere temperature and humidity differences has been roughly independent on the wind speed and can be observed in calm as well as in stormy conditions. Notably, south of ~50°S, these trends are almost everywhere accompanied by negative changes in the net longwave radiation (i.e., less longwave radiation leaving the ocean surface and/or more reaching it from the atmosphere), and by decreasing amounts of the shortwave radiation reaching the ocean (panels g,h in Fig. 5 and Supplementary Figs 7–11). All those factors consistently indicate that in the southern part of the domain of study, weather conditions with fog and/or low-level clouds have become more frequent. These findings agree with similar results obtained for global, monthly-averaged latent heat flux data^24^ and for annually-averaged data^18^.

At lower latitudes, the variance of (*T*_*s*_ −*T*_*a*_) and (*q*_*s*_ −*q*_*a*_) is larger, but in many regions the observations made above remain true, e.g., in the Indian Ocean (profile B, with negative trend in large (*q*_*s*_ −*q*_*a*_) differences at 30–40°S; Supplementary Figs 8 and 12d–f) and in the eastern Pacific (profile D, where the decrease of the 0.95-percentile of (*T*_*s*_ −*T*_*a*_) is uniform over all latitudes; Fig. 5a).

However, along the northern boundary of the domain of study, to the north of the ACC, the trends in 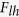 and, especially, 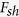, generally tend to be weak (Figs 2, 3, 4). At many locations they are opposite to those described above, e.g., along profiles C and E close to 40°S (Supplementary Figs 9,10), as well as along profile F, where, to the north of ~45°S, negative values of 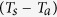 and small positive values of 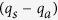 have become less frequent, leading to a roughly 40–50% decrease in very small and negative values of 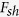 and, especially, 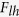 (Supplementary Figs 11, 14d–f and 15d–f).

## Is there periodicity in turbulent heat fluxes?

The existence of quasi-periodic variability in the extratropical atmosphere has been long predicted theoretically, but, contrary to the tropics, it wasn’t confirmed based on observational data until, very recently, Thompson and colleagues^25^,^26^ examined large-scale variability of the kinetic and potential energy in the extratropical Southern Hemisphere (20–70°S, 1000–200 hPa), and demonstrated that it is dominated by two distinct patterns, or structures: the (barotropic) southern annular mode (SAM), representing mainly the variance of the zonal-mean kinetic energy; and the baroclinic annular mode (BAM), representing the variance (rather than the mean) of the eddy kinetic energy and the eddy fluxes of heat^25^. The two patterns have been shown to be linearly independent and, most importantly, contrary to the red-noise spectrum of SAM, the spectrum of BAM has a clear and robust peak at periods in the range of 25–30 days. Similar periodicity has been found in the results of a number of numerical models with various degrees of complexity and in satellite-derived precipitation data^26^. What is particularly interesting from the perspective of this analysis, BAM has a clear signature in the variability of a number of lower-tropospheric phenomena, and correlates, e.g., with lower-tropospheric air temperature and anomalies of the eddy fluxes of heat at the 850 hPa level. Thus, it is reasonable to ask whether imprints of the BAM-specific periodicity can be found in the surface THF data. To this end, spectral analysis of the relevant variables was performed, in a way analogous to the one used in^26^ (see Methods), for zonally-averaged and spatially-varying time series. In both cases, a ‘peak-enhancement factor’ (or peak prominence), denoted with λ_*p*_, is used as an indicator of the presence and height of a spectral peak within the frequency range 0.031–0.04 day^−1^ (periods 25–32 days). As described in Methods, for spectra with power density decreasing with frequency—like those analyzed in this paper—values of λ_*p*_ lower than 1 can be expected if no peak is present in the defined range. Values considerably larger than 1 indicate the presence of a peak. Because this simple measure does not provide any information on the statistical significance, an additional analysis is performed, based on fitting a function to the spectrogram and checking whether the presumed peak lies above the upper 95% prediction bound of the fit (see Methods and Supplementary Note S2 for details).

**Figure 7:**
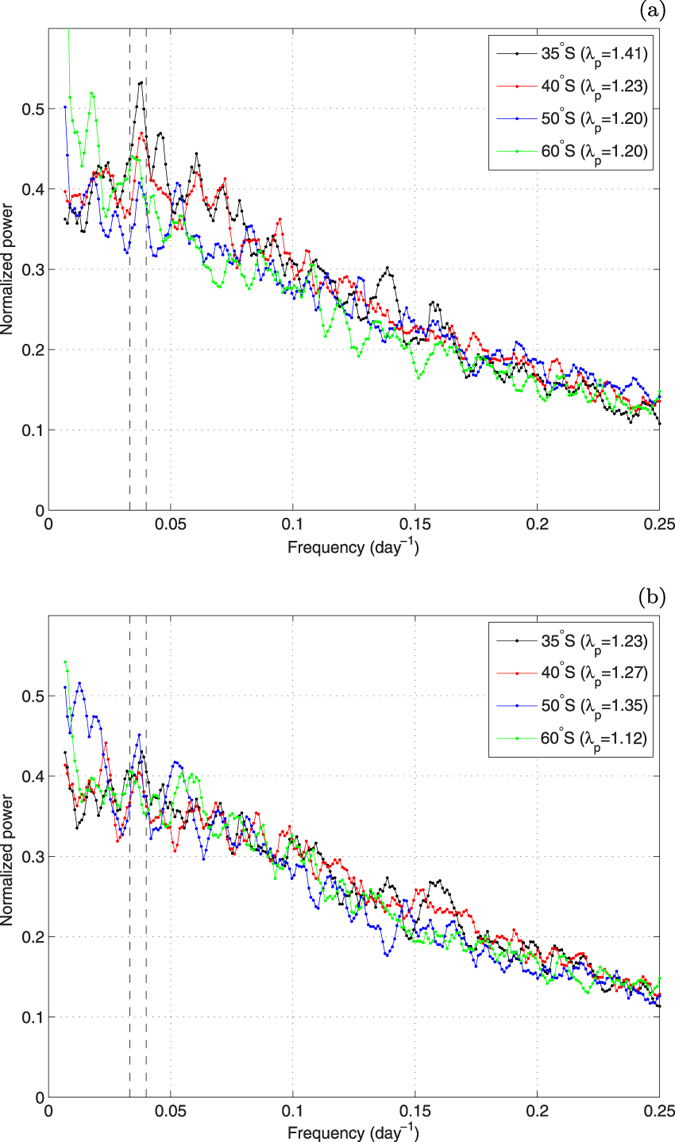
Normalized power spectra of zonally-averaged data at four selected latitudes for *F*_*lh*_ (a) and *F*_*sh*_ (b). Dashed vertical lines mark the range of frequencies corresponding to periods of 25–30 days. In (**a**), all peaks in this range lie above the upper 95% prediction bounds (Supplementary Fig. 16); in (**b**), only the peak for the 50°S-spectrum. See Supplementary Fig. 18 for corresponding spectra of other variables.

As shown in Fig. 7, spectral peaks do exist in the zonally averaged THF data, especially northward from 40°S, where there is a clear global maximum of the spectral power within the frequency range in question. The peaks lie above the 95% prediction bounds in the case of all spectrograms of 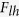 (i.e., at all latitudes; Supplementary Fig. 16), and around the 50°S latitude in the case of the spectrograms of 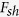. The presence of this periodic signal derives mainly from the surface wind, although at some latitudes it is visible in the air temperature and humidity data as well (Supplementary Fig. 18a–c). Contrary to the power spectra of 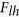, 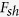, 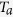, 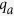 and 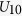, which exhibit approximately exponential decrease of the power density with frequency, the spectra of 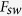 and 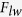, 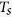, and thus those of 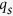, are of power-law type and do not have any significant peaks within the frequency range of interest (Supplementary Fig. 18d–f). Whereas this could be expected in the case of 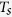, it is rather surprising in the case of the radiative fluxes in view of the fact that they depend on processes taking place not only within, but also above the ABL, where the signature of BAM could be expected to manifest itself, e.g., in cloudiness (as already mentioned, it is present in precipitation data).

**Figure 8:**
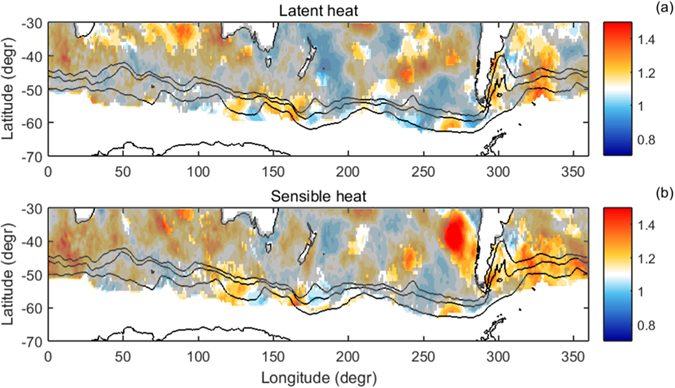
Maps of the peak-enhancement factor λ_*p*_ for the spectra of *F*_*lh*_ (a) and *F*_*sh*_ (b). Regions where the spectral peaks within the BAM frequency range do not exceed the upper 95% prediction bounds are shaded. See Supplementary Fig. 19 for corresponding maps of λ_*p*_ for the spectra of other variables.

The strength of the periodic signal is not zonally uniform, and the spatial variability of λ_*p*_ (Fig. 8) in many respects resembles the spatial signature of BAM in the air-temperature variance (Fig. 10d in ref. 25 and Fig. 1c in ref. 26). The highest values of λ_*p*_ occur in the southern Atlantic, eastern Pacific, as well as some parts of the Indian Ocean, and the results of the exponential fit to the data suggest that the corresponding peaks are statistically significant in these regions. Some of the spots of large λ_*p*_ values result from analogous spots of λ_*p*_ for 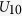, other from those for 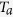 and/or 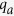 (Supplementary Fig. 19). Importantly, very similar spatial patterns of λ_*p*_ and shapes of the zonally-averaged spectra were obtained from another (NCEP-DOE) data set (Supplementary Fig. 17), reinforcing the robustness of the results.

## Discussion

Among the main findings of this study is, firstly, that in most areas of the Southern Ocean, statistically significant trends in the moments of the THF pdfs are observed in the analyzed time period, and secondly, that these trends regard the shape and width as much as the central tendency of the pdfs. (Notably, the central tendency—or, specifically, the mean—is the only quantity analyzed in studies based on seasonally/annually averaged data^16^,^18^). Among the consequences of these changes is the decreasing occurrence probability of extreme ocean–atmosphere heat flux events, observed over large parts of the Southern Ocean. Such events occur frequently around SST fronts associated with the western boundary currents, but, although they play a very important role in the evolution of the oceanic mixed layer, little is known about their frequency and typical magnitude in the Southern Ocean^15^. They may occur at exceptionally high wind speeds (particularly frequent in the Indian Ocean sector of the Southern Ocean^23^), but are predominantly associated with advection of air masses with different temperature and/or humidity—at any selected location, the time scale of the variability of 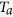 and 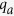 is much shorter than that of 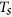 (and, consequently, 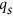). Hence, 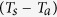 is strongly negatively correlated with 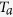, and correlation between 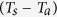 and 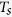 is statistically insignificant—as has already been noticed for the SOFS site (magenta point in Fig. 1)^15^. Consequently, 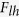 and 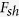 are strongly negatively correlated with 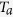 (especially within and to the north of the ACC), while the corresponding correlation with 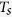 is insignificant almost everywhere in the study domain and rarely exceeds the value of 0.2 (Supplementary Figs 20 and 21). Notably, the correlation with 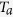 decreased (in terms of its absolute values) in the analyzed period: the differences between the first and last decade are highest within the Agulhas Return Current, but occur over large parts of the study domain, with an exception of the region to the east and south-east of New Zealand. This means that other factors influencing THF have become more important during that period. Although, as already mentioned, correlations with 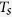 are very small, they have increased throughout the whole period and changed sign from negative to positive to the north of ACC in the Indian Ocean and in the ACC loop in the south-west Atlantic (Supplementary Fig. 21). Characteristically, these two regions have witnessed very strong (20–40%) positive trends in the variance of the meridional sea surface temperature gradient 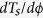 (Supplementary Fig. 22). In order to analyze the role of this variable in more detail, wind direction data would be necessary (for example from Quikscat or another similar data source), as 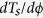 can be expected to influence the THF in situations with large meridional wind speed component, when strong advection prevents the air temperature from adjusting to the temperature of the underlying ocean. At present, we can only speculate that the increased variance of the cross-frontal temperature differences has been at least partly responsible for the lack of a negative trend in large 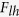 and 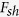 values, that can be seen in the affected zones along profiles A, B, E, and F.

Outside of these regions, as described earlier, the trends observed in the properties of the analyzed pdfs indicate that the occurrence probability of extreme heat flux events decreased in the analyzed time period, as the pdfs narrowed and became more skewed (Figs 3 and 4). Accordingly, the return period of high-THF events increased and the 0.95-quantile decreased almost everywhere to the south of ~45°S (except in some areas E and SE of New Zealand; not shown).

The findings presented here are particularly interesting in view of the fact that one of the consequences of the recent climate change in the Southern Hemisphere is an already-mentioned intensification of the circumpolar-vortex circulation, a poleward shift of the westerlies, and an associated increase in surface wind speeds^27^. Climate models predict that this trend is likely to continue into the following decades^28^. These trends could suggest a simple, but, as it seems, premature conclusion that the amplitude of the turbulent heat fluxes over the affected ocean regions should increase as well, through their direct dependence on 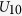 (Eqs 1 and 2). However, if the trends present in the OAFlux data are correct, the opposite is the case due to the prevailing effect of decreasing air–water temperature and humidity differences.

**Figure 9:**
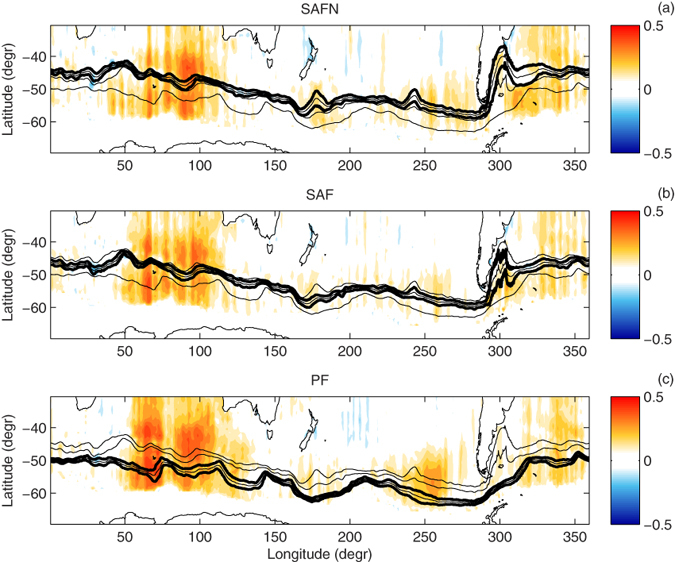
Maps of the lagged correlation coefficient between *F*_*lh*_ and the position of the three main ACC fronts: SAFN (**a**), SAF (**b**) and PF (**c**). Thin black lines show the main position of the three ACC fronts; thick black lines mark the distance of one standard deviation from the mean position of the front analyzed in the respective map. See Methods for details, and Supplementary Fig. 23 for corresponding maps for 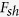.

Another important aspect of this analysis concerns relationships between the THF variability and changes in the positions of the major fronts within the ACC. Details of the response of the oceanic fronts to the changing atmospheric circulation are at present not well understood. The results of various observational and modeling studies are not conclusive and very sensitive to the spatial and temporal resolution of the data, as well as the time period and region analyzed. Generally, the results of the low-resolution global climate models show a strengthening and a poleward shift of the ACC in response to changes in the atmospheric forcing^29^. Studies based on higher-resolution simulations sufficient to resolve the details of the ocean bottom topography suggest a more complicated oceanic response, with barotropic and baroclinic fronts reacting to changing winds in a different way: whereas baroclinic fronts, characterized by strong horizontal temperature gradients and confined to the upper layers of the ocean, adjust their position to changing wind stress patterns, barotropic fronts extend deep into the water column and their position, constrained by bottom topography, remains stable in a changing climate, especially in regions with steep bottom slopes^22^,^30^. All this indicates that the ocean–atmosphere heat flux can be expected to respond differently to the changing atmospheric circulation in regions where the fronts shift their positions than in regions where they are stationary. A lagged correlation analysis (see Methods) shows that there exist distinct regions in the Southern Ocean where the THF is correlated with the latitude of the ACC fronts (Fig. 9 and Supplementary Fig. 23): the Indian Ocean, the south-western Atlantic, the south-eastern Pacific, and a small area south of New Zealand, to the east of the Campbell Plateau. Understandably, the correlation is insignificant in regions where the fronts are constrained by topography and their position remains stable over time (Supplementary Fig. 24), e.g., at 140–160°E, where the ACC crosses the Southeast Indian Ridge (Fig. 1), or at 200–230°E, where it is influenced by the Eltanin Fracture Zone. The correlation tends to be higher in regions with strong variability of the frontal positions, like, e.g., in the lee of bathymetric features. The differences between the two types of behavior of the fronts are clearly visible at the Kerguelen Plateau (~70°E). Whereas SAF and SAFN converge and flow around the shallow zone to the north of it, the PF passes over it (what Sallée and colleagues^30^ call the merging and shoaling effect, respectively). Consequently, the variance and trend of the SAF and SAFN positions are close to zero, while that of the PF are among the highest in the whole ACC (Supplementary Fig. 24), and thus the PF position explains a clearly higher part of the THF variance, and over a larger area, than the positions of SAF and SAFN (Fig. 9 and Supplementary Fig. 23). Analogously, in the lee of the Campbell Plateau the correlations are highest for SAFN, which has the largest variability of the three fronts. In summary, the interactions of the ACC with bottom topography influence the ocean–atmosphere heat exchange. Notably, the centers of the highest correlation between the THF and the fronts’ positions coincide with regions of significant correlation between those positions and the SAM index at short time scales (below 3 months)^30^.

Overall, the results presented in this paper demonstrate the complex, intricate nature of relationships between the ocean–atmosphere turbulent heat flux and other components of the climate system. Many aspects of these relationships manifest themselves at short, daily and synoptic time scales and cannot be analyzed based on monthly or seasonal data. Our better understanding of these short-time processes is important as at least some of them may have a considerable influence not only on the local weather, but also on the climate of the whole Southern Hemisphere.

## Methods

### Data sources

The OAFlux products were provided by the Woods Hole Oceanographic Institution (WHOI) OAFlux project funded by the NOAA Climate Observations and Monitoring (COM) program^17^. For details on the ISCCP data, see http://isccp.giss.nasa.gov/. Both OAFlux and ISCCP datasets are available through the OAFlux home page at http://oaflux.whoi.edu/. The weekly positions of the three major ACC fronts from the AVISO program^30^ are available at http://ctoh.legos.obs-mip.fr/. The Southern Ocean Flux Station (SOFS) data are made available through the home page of the Integrated Marine Observing System (http://imos.aodn.org.au/imos/). For the sources of other data sets, used in the additional analyses (intercomparison of THF products) and presented in the Supplementary Material, see references there.

### Uncertainties in the OAFlux data

The quality assessment of THF data in remote regions of the Southern Ocean is difficult due to very limited availability of observational data (especially from the cold season) that can be used for validation. Regular measurements at oceanic mooring sites are almost exclusively limited to the tropics and subtropics. Not surprisingly, inter-comparisons of various products^7^,^17^,^18^,^20^,^24^,^31^,^32^,^33^ reveal significant discrepancies between data obtained from different sources. The estimated uncertainties of the OAFlux products are provided with the data^17^. They were calculated based on available ship observations and other, mostly incidental data sources. However, from the point of view of this work, which concentrates on the pdfs of the analyzed variables, systematic, long-term measurements would be necessary, sufficient to estimate observed pdfs. Here, observational data from the Southern Ocean Flux Station (SOFS) are used. SOFS—a moored buoy located within the ACC at 142°E, 46°45’S (magenta point in Fig. 1)—is the first deep-water station providing continuous oceanographic and meteorological measurements in the Southern Ocean^15^. At present, the data are available in the periods 17 Mar 2010–13 Mar 2011, 1 Jan–6 Mar 2012 and 14 Jul–6 Nov 2012. The results of the validation are presented and discussed in Supplementary Note S1.1. Additionally, intercomparisons of the OAFlux data and three other data sources are performed, and for one of these products analogous validation with the SOFS data is conducted (the remaining two are unavailable in the time period of SOFS data)—see Supplementary Note S1.2.

### Data analysis

This study concentrates on the extratropical Southern Hemisphere ocean south of the 30°S. The analysis is performed for all sea points that remained ice-free during at least 70% of the time in the whole analysis period 1985–2013, i.e., at least during 7415 days (see dashed line in Fig. 1). The probability density functions (pdfs) of the analyzed quantities were estimated for 10-year-long periods with a moving-window method, from 1985–1994 up to 2004–2013. An example of the time series of pdfs of 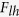 and 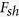 obtained in this way is shown in Supplementary Fig. 6 for an arbitrary selected point within the area of study. It is representative for the whole dataset in that it exhibits a uniform, consistent trend throughout the whole analysis period.

The pdfs from the two outermost decades were compared by means of a standard two-sample Kolmogorov–Smirnov test, based on the null hypothesis 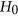 stating that the samples are from the same continuous distribution. The pdfs were regarded as different if the test rejected 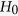 at the 1% significance level. The differences of the analyzed moments—median, standard deviation and skewness (third standardized moment)—of the pdfs were analyzed only in points in which 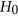 could be rejected (the remaining points are left blank on the maps of differences in Figs 2, 3, 4).

The meridional profiles A–F (thick lines in Fig. 1) were selected so that they run through regions exhibiting some characteristic, important aspects of the data. Profiles A and B cross the Agulhas Retroflection. Profile C runs through the location of the SOFS station. Profile D crosses the south-east Pacific Ocean in region with strong variability of the position of the ACC fronts, and the spot of high λ_*p*_ values to the west of the coast of South America; Profile E runs through the Drake Passage. Profile F crosses regions directly to the east of the Drake Passage, as well as the loops of the ACC currents to the east of the South American continent.

The power spectra of the analyzed variables were estimated for linearly-detrended time series by means of the short-time Fourier transform method, in a way analogous to that of Thompson and Woodworth^25^. The signal was divided into 513 segments (with the segment overlap equal to 500), the length of the Hanning window and the FFT length equaled 1024. The average of the resulting 513 spectrograms was then smoothed with a 5-point running mean in order to obtain the final spectra, including those presented in Fig. 7 and Supplementary Fig. 18.

The peak-enhancement factor λ_*p*_ is defined as a ratio between the maximum spectral power within the frequency range 0.0313–0.0391 day^−1^ to the minimum spectral power within the frequency range 0.0166–0.0293 day^−1^. The first range corresponds to periods ~25–32 days (the “BAM range”), the second—to periods ~34–60 days. For a spectrum with power density decreasing with frequency, λ_*p*_ < 1 is expected. Tests with an alternative definition of λ_*p*_, based on mean values of the spectral power instead of the min/max values, gave very similar results, although the numerical values of λ_*p*_ were slightly different. In order to obtain some estimate of the statistical significance of the peaks, a continuous (exponential or power-law, depending on the analyzed variable) function was fitted to the individual spectrograms, and the height of the peaks above/below the 95% upper prediction bounds was determined—see Supplementary Note S2 for a discussion and details of the procedure. In the maps of λ_*p*_ (Fig. 8 and Supplementary Figs 17 and 19), regions where the “BAM peaks” do not exceed that level are shaded.

Figure 9 and Supplementary Fig. 23 show the lagged Pearson correlation coefficient between 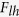, 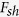 and the latitude position of the PF/SAF/SAFN fronts, for THF lagging the front positions. For each point on the given map, the figure shows the maximum value of the correlation coefficient obtained for lags in the range 0–50 days. At roughly 73% of all points with correlation coefficient exceeding 0.25 (orange and red regions on the maps), the maximum correlation occurred for lags lower than 10 days. As can be seen, in the remaining (white) regions no significant correlation could be found even for lags as high as 50 days. The THF data were smoothed with a moving-average method (window length equal 7 days) before calculating the correlation.

## Additional Information

**How to cite this article**: Herman, A. Trends and variability of the atmosphere–ocean turbulent heat flux in the extratropical Southern Hemisphere. *Sci. Rep.* **5**, 14900; doi: 10.1038/srep14900 (2015).

## Supplementary Material

Supplementary Information
