# Supplementary material for: Trends and variability of the atmosphere–ocean turbulent heat flux in the extratropical Southern Hemisphere
Source: Sci Rep. 2015 Oct 9;5:14900. doi: 10.1038/srep14900 (PMC4598877; doi:10.1038/srep14900)
Supplement: Supplementary Information [file srep14900-s1.pdf]

Supplementary Material to

"Trends and variability of the atmosphere–ocean turbulent heat flux in the extratropical Southern Hemisphere"

Agnieszka Herman

Institute of Oceanography, University of Gdansk, Poland
Email: oceagah@ug.edu.pl

Supplementary Note S1

Validation of monthly, seasonal and/or annual OAFlux data and its performance in comparison with other THF data sets, has been presented in a number of papers [1–9]. In particular, the OAFlux products have been shown to compare favorably with buoy measurements in comparison to reanalysis products (ERA-40 and NCEP). However, all buoys used in the validation were situated north of 30°S, i.e., outside of the domain of study of this work.

Here, in Section S1.1, we perform a comparison of the OAFlux data with observations from the Southern Ocean Flux Station (SOFS) – which, to the best of my knowledge, has not been published before – with full awareness that the results of this comparison, based on data from a single point within a very large domain, have mainly an illustrative purpose and cannot be generalized to other locations.

Further, Section S1.2 discusses the results of comparisons between the OAFlux data and selected other turbulent heat flux datasets:

- NCEP-DOE:
  the National Centers for Environmental Prediction NCEP-DOE Reanalysis 2 [10], available on a global grid with resolution of ∼1.9° (for surface variables), 6-hourly within the period 1 Jan 1979 until present; here used in the period 1985–2013, the same as for OAFlux,
- GSSTF.3:
  the Goddard Satellite-based Surface Turbulent Fluxes dataset, version 3 [11], available on a global grid with resolution of 0.25°, daily within the period 9 Jul 1987–2 Dec 2008;
- HOAPS:
  the Hamburg Ocean Atmosphere Parameters and fluxes from Satellite data, version 3.2 [12,13], available on a global grid with resolution of 0.5°, daily within the period 1 Jul 1987–31 Dec 2008.

Again, contrary to earlier works, the analysis concentrates on daily, short-term variability of the THF components.

It is worth noting that the HOAPS and GSSTF.3 data sets are based exclusively on satellite data sources, and consequently contain gaps, especially in the northern part of the domain of study (roughly 15% of the time, as compared to 5–10% in its central parts).

S1.1. Validation of the OAFlux products against SOFS data

The SOFS data are available in the periods 17 Mar 2010–13 Mar 2011, 1 Jan–6 Mar 2012 and 14 Jul–6 Nov 2012. (The buoy has been successfully re-launched in March 2015.) Prior to further analysis, daily averages of the data were calculated for days with at least 12 individual measurements. This resulted in time series of length $N = 524$. Supplementary Table 1 summarizes the results of the basic comparative analysis between the OAFlux and the SOFS meteorological/hydrological data and the turbulent flux components (unfortunately, the SOFS measurements lie outside of the data coverage of the ISCCP data set – which ends in 2009 – and hence no analogous comparison for the radiative flux data can be made). Fragments of the analyzed time series in the period 17 Mar 2010–13 Mar 2011 are shown in Supplementary Fig. 1. In the table, r^2 denotes the Pearson correlation coefficient and σ – standard deviation of differences. Supplementary Fig. 2 compares the cumulative distribution functions from the SOFS and OAFlux data set.

As can be seen, the OAFlux air-temperature values are slightly higher than the measured ones almost throughout the whole analysis period, with a positive bias of 0.42°C. This manifests itself in a negative bias in F_{sh}, as F_{sh} is strongly negatively correlated with T_a (see further Supplementary Fig. 20).

The range of variability of q_a in the OAFlux data is underestimated, similarly as the range of variability of F_{lh}, which is a function of the air humidity. No significant biases or deviations are present in the wind speed data – the pdfs of U_{10} are almost identical (very high p-values of the K-S test).

Overall, at the SOFS location the OAFlux latent heat flux tends to be underestimated within the range of low values, and overestimated within a range of high values (Supplementary Fig. 2e).

Noticeably, both datasets show a seasonal cycle in both air and water temperature, as well as in the air humidity, but no seasonal cycle is present in the turbulent heat flux time series, dependent on the difference $T_s - T_a$ and $q_s - q_a$ (Supplementary Fig. 1). In the case of the THF, short-term, synoptic variability dominates over longer-term, seasonal signal.

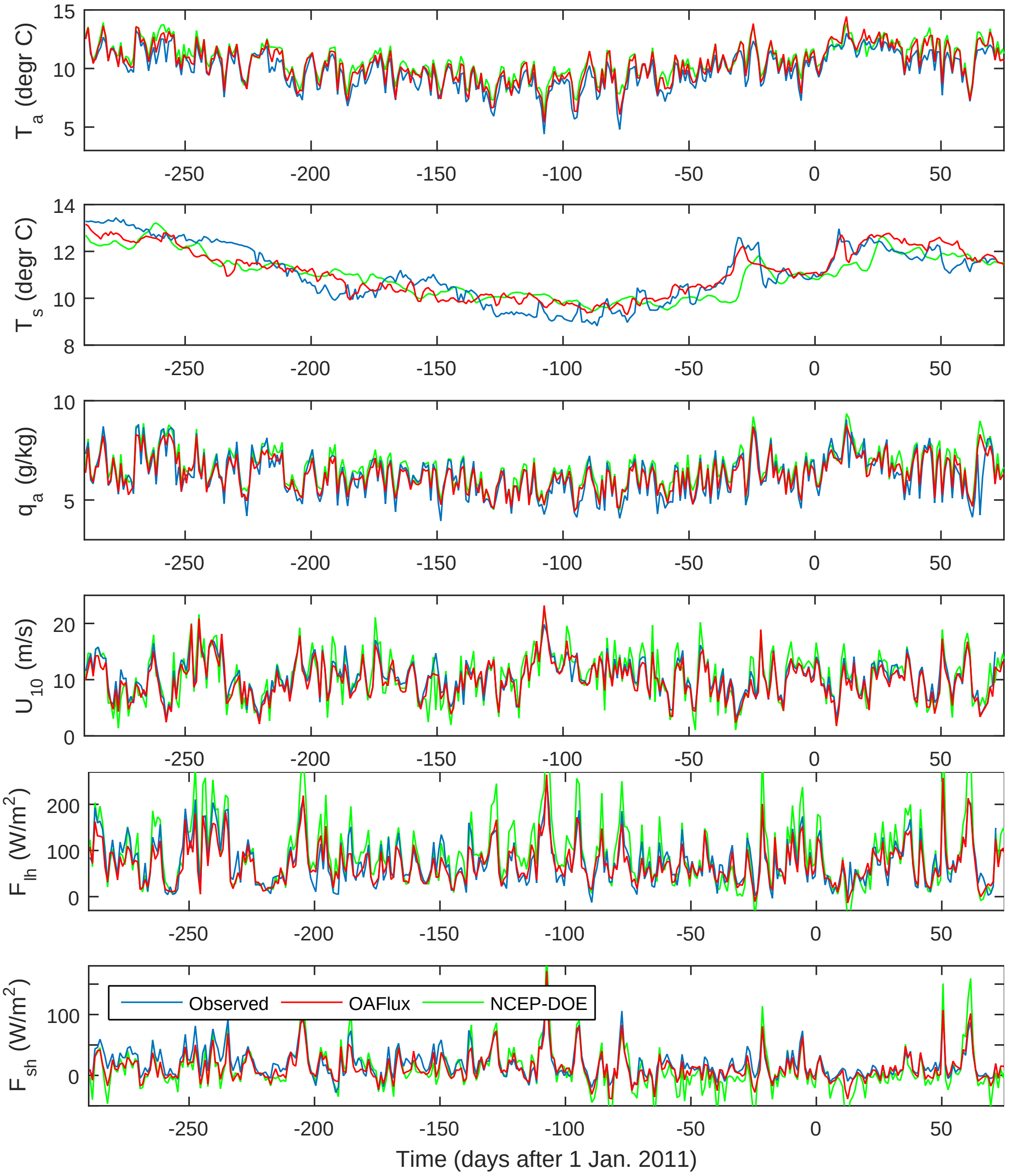

Supplementary Figure 1: Time series of the meteorological variables (T_a, T_s, q_a, U_{10}) and the turbulent heat flux (F_{lh}, F_{sh}) at the SOFS station (magenta point on the map in Fig. 1 in the main text) in the period 17 Mar 2010–13 Mar 2011: measured (blue), from the OAFLux data set (red), and from the NCEP-DOE data set (green).

Supplementary Table 1: Comparison between the SOFS and OAFlux data

Variable	r^2	σ	bias	K-S test p-value
T_a	0.97	0.42°C	0.42°C	0.004
q_a	0.90	0.48 g/kg	−0.01 g/kg	0.046
T_s	0.91	0.52°C	0.10°C	$2{\cdot}10^{-7}$
U_{10}	0.90	1.49 m/s	0.07 m/s	0.736
F_{sh}	0.88	11.23 W/m^2	−3.94 W/m^2	0.013
F_{lh}	0.88	22.48 W/m^2	0.42 W/m^2	0.003
$T_s - T_a$	0.86	0.61°C	−0.32°C	0.004
$q_s - q_a$	0.80	0.57 g/kg	0.02 g/kg	0.017

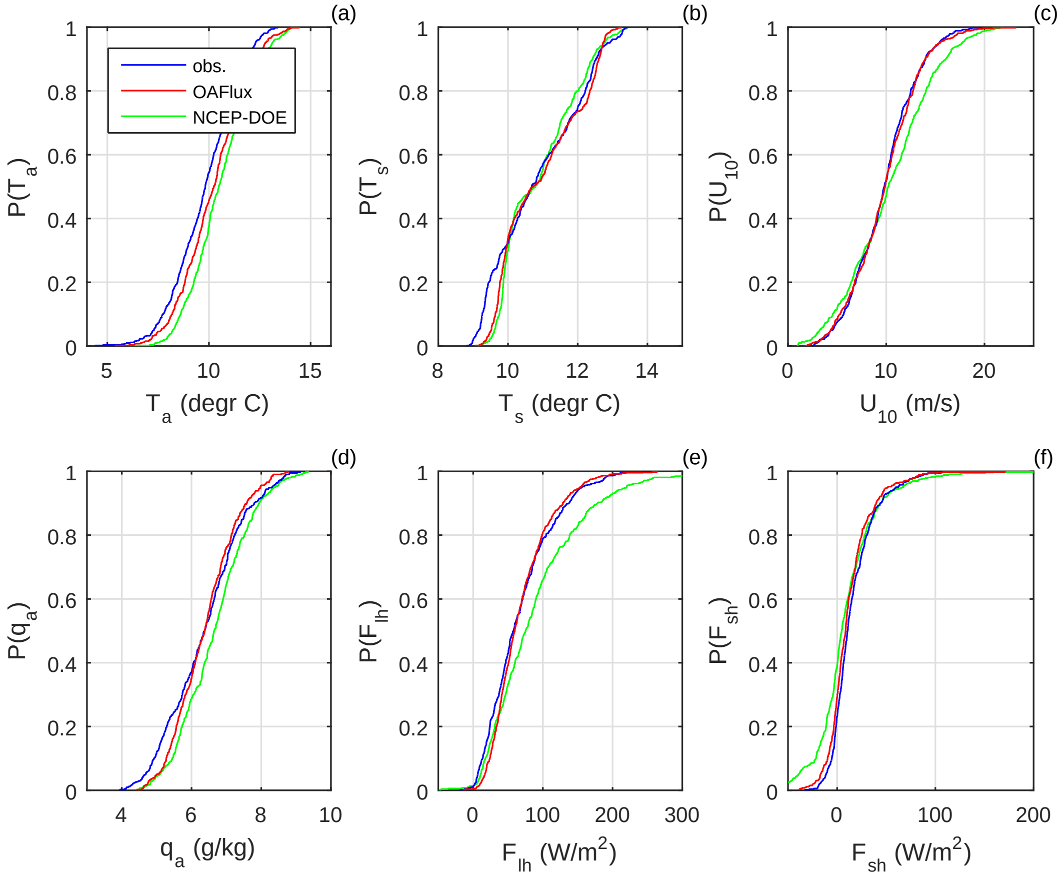

Supplementary Figure 2: Cumulative distribution functions of the meteorological variables (T_a, T_s, q_a, U_{10}) and the turbulent heat flux (F_{lh}, F_{sh}) at the SOFS station (magenta point on the map in Fig. 1 in the main text): measured (blue), from the OAFLux data set (red), and from the NCEP-DOE data set (green).

S1.2. Comparison between different data sets

Unfortunately, the satellite-data-based GSSTF.3 and HOAPS products are available only until the end of 2008 (see the list in the introduction to this note) and therefore cannot be validated against the SOFS measurements. The results of validation of the NCEP-DOE data are shown in Supplementary Figs. 1, 2 and Supplementary Table 2.

The time series in Supplementary Fig. 1, and the cdfs in Supplementary Fig. 2e,f show that the most evident feature of the NCEP-DOE data is overestimation of the extremes, especially positive extremes of F_{lh} and negative extremes of F_{sh}. Both effects are strongly related to overestimated variability of wind speed (the standard deviation of differences σ between the modeled and measured wind speed reaches almost 2 m/s), as well as to bias in air temperature and humidity (0.78°C and 0.31 g/kg, respectively). As a result, almost all statistics describing the accuracy of F_{lh} and F_{sh} are considerably worse for NCEP-DOE than for OAFlux. In particular, whereas the bias for both OAFlux THF components is negligible, it exceeds 21 W/m^2 for the NCEP-DOE latent heat flux. The standard deviation of differences of both NCEP-DOE THF products is more than 60% higher than for analogous OAFlux products.

In an attempt to assess the quality of the other two data sources, GSSTF.3 and HOAPS, we first determine the cdfs of F_{lh} and F_{sh} from the OAFlux data for three different time periods: 2004–2008, 2009–2013, and the third one corresponding to the period of availability of the SOFS observational data (exactly as analyzed before). Supplementary Figs 3d and 4d show that the respective cdfs hardly change, suggesting that, first, the data from the "SOFS-period" are representative for a longer time period spanning 5 years, and second, there is no significant trend in the OAFlux fluxes between 2004–2008 and 2009–2013. Combined with the above-demonstrated high quality of the OAFlux product at the SOFS station, we may formulate a hypothesis that the F_{lh} and F_{sh} from OAFlux in the years 2004–2008 reflect the real-world variability of these variables at the analyzed location. We may then compare the other products to OAFlux in period 2004–2008 (in which all four data sets are available), as shown in Supplementary Figs 3 and 4.

Among the four data sets, the slope of the cdf of F_{lh} is steepest in the case of OAFlux, i.e., the range of variability of these data is the lowest. The scatterplots in Supplementary Fig. 3b,c show also that the F_{lh} values of the two satellite-derived products are limited from below (by zero in HOAPS and -18 W/m^2 in GSSTF.3). The shapes of the clouds of dots suggest that the algorithms produce values lower than these limits which are then adjusted to the allowed range. In the case of F_{sh}, the HOAPS and, especially, GSSTF.3 cdfs agree well with that of OAFlux within the range of negative values, whereas for positive F_{sh} only the NCEP-DOE cdf is close to that of OAFlux (Supplementary Fig. 4d). Here, again, the range of variability of the NCEP-DOE data is the largest among the four data sets; it is smallest in the case of GSSTF.3, where values larger than ± 50 W/m^2 occur only sporadically.

The lack of observational data from other locations makes it impossible to assess the quality of the four datasets in other parts of the domain of study. However, it is worth noting that the relationships between different THF products are similar everywhere – as the histograms of F_{lh} and F_{sh} in Supplementary Fig. 5 clearly show. They were calculated for pairs of values from the whole data sets, i.e., each histogram represents over 65 millions of points from the whole domain and from the period 1987–2008, common to all data sets. They have analogous features to those discussed above (note that the color scale is logarithmic, so that the scatter of points seems larger than it really is). In summary: (i) the NCEP-DOE F_{lh} product has larger variance than that of the other products, due to higher extremes; (ii) the deviations between the OAFlux and NCEP-DOE F_{lh} are smaller than those between OAFlux and the remaining two products; (iii) the values of F_{lh} in HOAPS and GSSTF.3 data are cut at the prescribed lower limit; (iv) the values of the NCEP-DOE F_{sh} are similar to those from OAFlux for $F_{sh} > 0$, but considerably larger (in terms of amplitude) for $F_{sh} < 0$; (v) the differences between the pairs of F_{sh} products are largest for OAFlux–HOAPS data; (vi) contrary to the remaining products, the values of the HOAPS F_{sh} are never smaller than -50 W/m^2, presumably due to some "limiter"; (vii) the range of variability of the GSSTF.3 F_{sh} is

Supplementary Table 2: Comparison between the SOFS and NCEP-DOE data

Variable	r^2	σ	bias	K-S test p-value
T_a	0.93	0.57°C	0.78°C	$1{\cdot}10^{-6}$
q_a	0.90	0.46 g/kg	0.31 g/kg	$3{\cdot}10^{-4}$
T_s	0.85	0.69°C	−0.01°C	0.002
U_{10}	0.84	1.97 m/s	0.11 m/s	0.11
F_{sh}	0.83	18.24 W/m^2	−6.88 W/m^2	$9{\cdot}10^{-10}$
F_{lh}	0.87	34.43 W/m^2	21.60 W/m^2	0.004
$T_s - T_a$	0.79	0.61°C	−0.32°C	$1{\cdot}10^{-18}$
$q_s - q_a$	0.80	0.72 g/kg	−0.79 g/kg	$3{\cdot}10^{-4}$

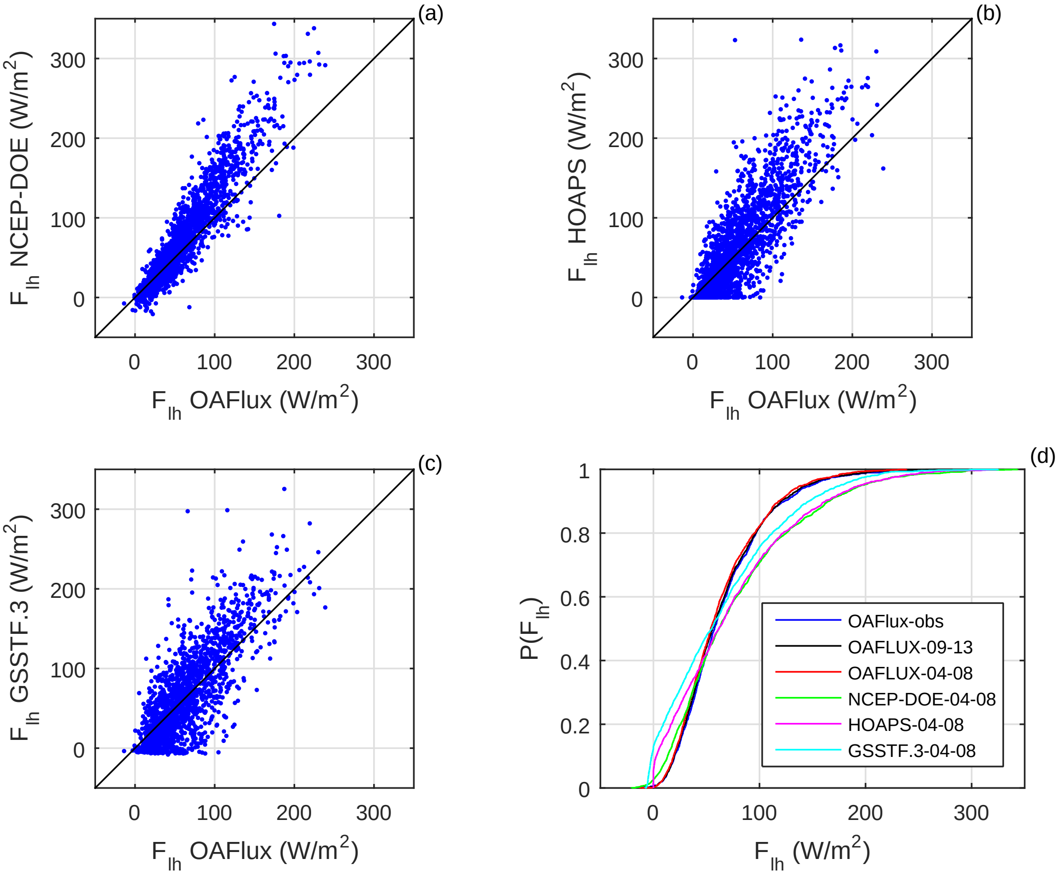

Supplementary Figure 3: Comparison of the analyzed F_{lh} products at the SOFS station. Panels (a–c) show scatterplots between the OAFlux data and NCEP-DOE (a), HOAPS (b), and GSSTF.3 (c) from the period 2004–2008. Panel (d) shows the cumulative distribution functions of F_{lh} for all datasets from the period 2004–2008, and for the OAFlux data from two additional time periods: 2009–2013 and that in which observational data are available (as in Supplementary Fig. 2e).

considerably lower than that of the OAFlux data.

Additional analysis (not shown) confirmed that these observations have location-independent character. For example, the ratio of the variance of F_{lh} between the OAFlux and NCEP-DOE products is lower than 1 everywhere in the domain of study; similarly, the ratio of the variance of F_{sh} between the OAFlux and GSSTF.3 is everywhere higher than 1.

Obviously, the above-described observations cannot be used to justify statements concerning the quality of the analyzed data sets in different parts of the area of study. The existence of certain general relationships between the particular data sets presumably results from certain assumptions and parameterizations underlying the algorithms, different types/sources of data used as input, etc. However, the performance of those algorithms, and thus the quality of the final products, may be spatially variable, and validation at one location – the SOFS station – remains insufficient to estimate that quality elsewhere.

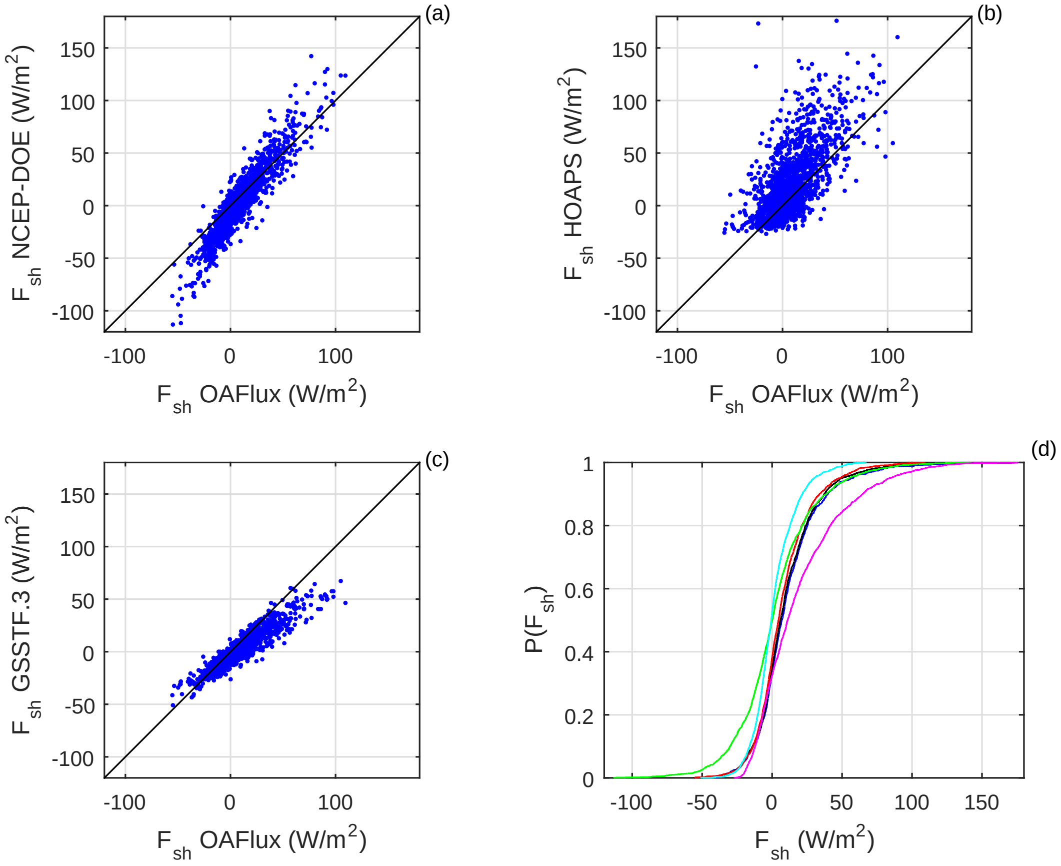

Supplementary Figure 4: As in Fig. 3, but for F_{sh}.

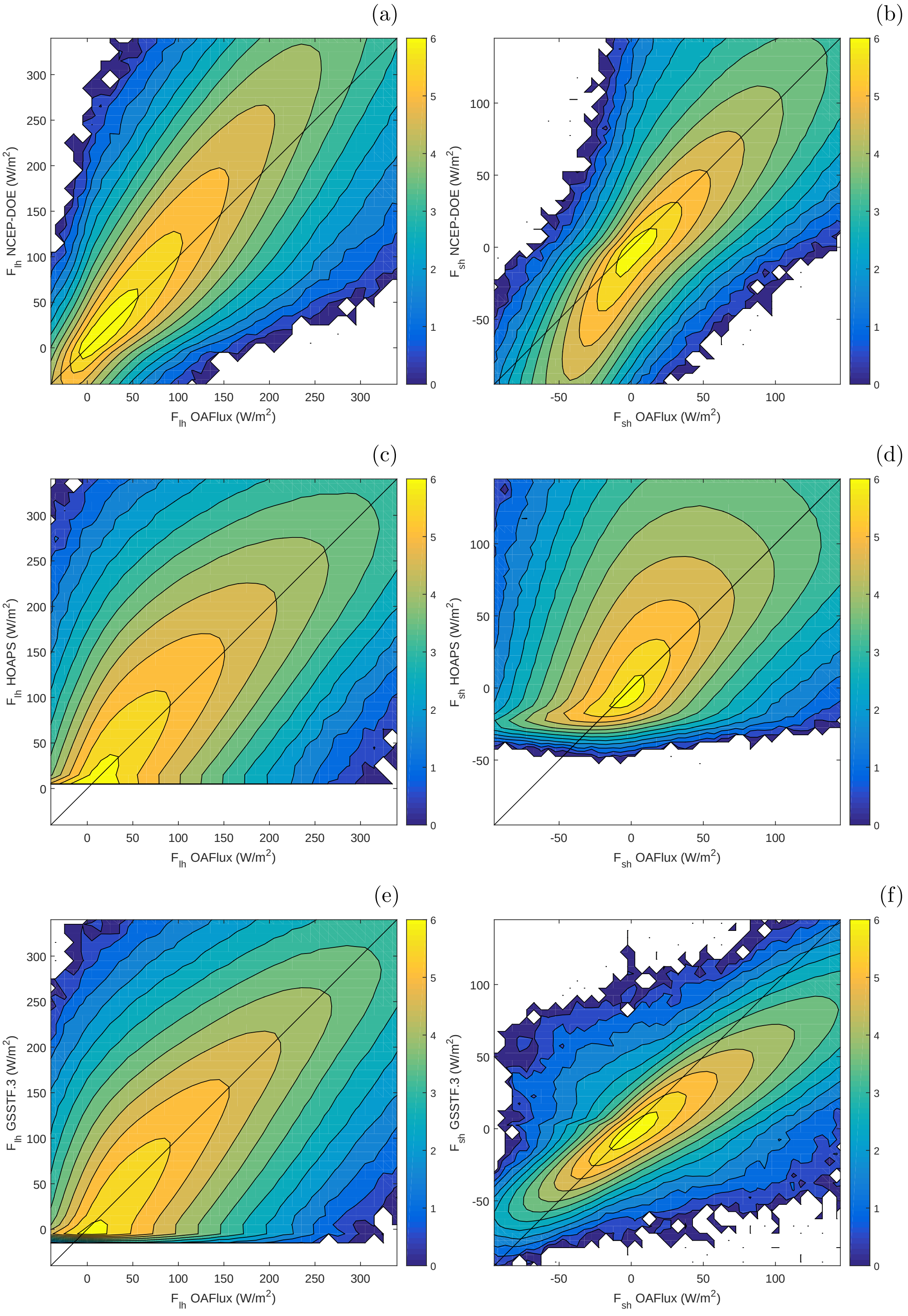

Supplementary Figure 5: Histograms of F_{lh} (a,c,e) and F_{sh} (b,d,f) for all data (i.e., the whole domain) from the period 1987–2008: OAFlux *versus* NCEP-DOE (a,b), HOAPS (c,d), and GSSTF.3 (e,f). The color scale is logarithmic. White areas correspond to no-data bins. Bin width equals 10 W/m^2 for F_{lh} and 5 W/m^2 for F_{sh}.

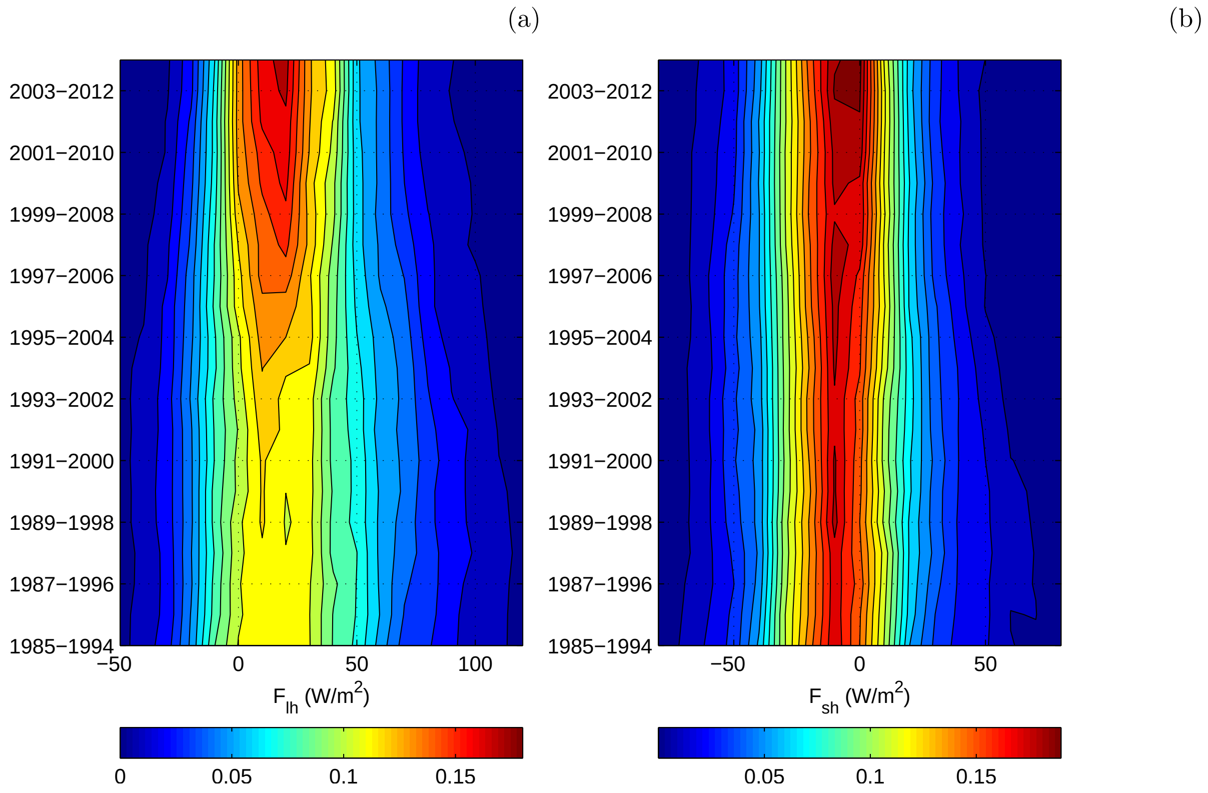

Supplementary Figure 6: Temporal evolution of decadal pdfs of F_{lh} (a) and F_{sh} (b) in a point at 48°E, 55°S.

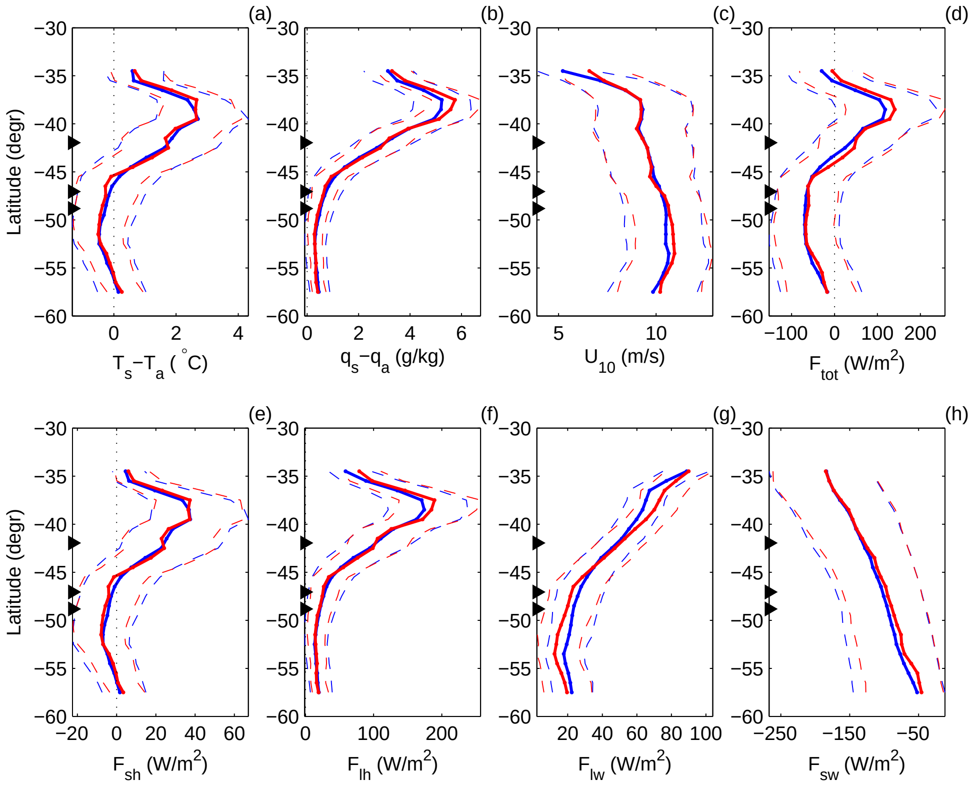

Supplementary Figure 7: Changes of the analyzed variables (a: temperature difference $T_s - T_a$; b: humidity difference $q_s - q_a$; c: wind speed U_{10}; d: total heat flux F_{tot}; e–h: sensible F_{sh}, latent F_{lh}, longwave F_{lw} and shortwave F_{sw} heat flux, respectively) along a meridional profile at 22°E (line A on the map in Fig. 1): median values (thick continuous lines), 0.25 and 0.75 quartiles (thin dashed lines) in the first (1985–1994; blue) and last (2003–2012; red) decade of the analyzed period. Black triangles mark the average positions of the PF, SAF and SAFN. Note that F_{lw}, F_{sw} and F_{tot} in the second time period are limited to 2003–2009. See also Fig. 5 in the main text.

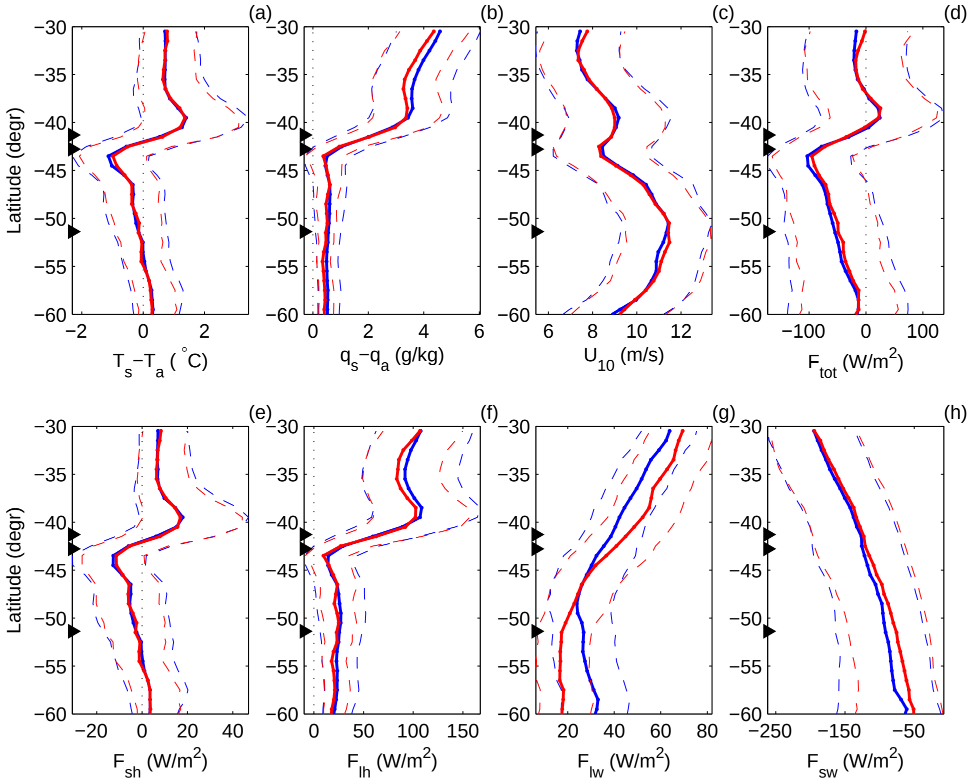

Supplementary Figure 8: As in Supplementary Fig. 7, but for a profile at 48°E (line B on the map in Fig. 1 in the main text).

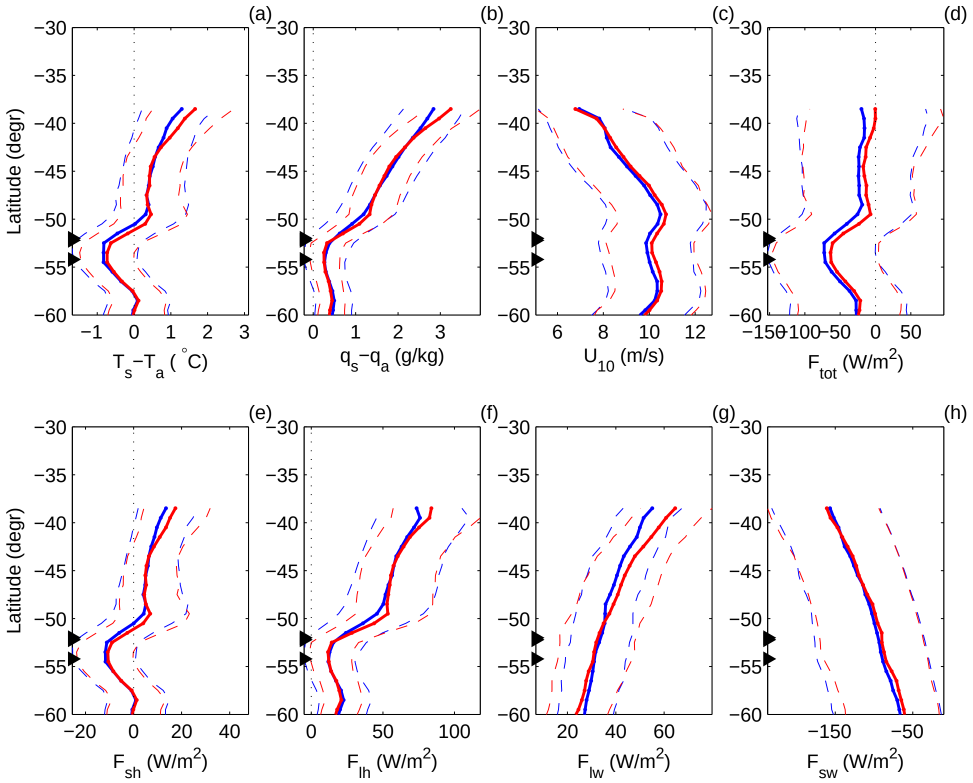

Supplementary Figure 9: As in Supplementary Fig. 7, but for a profile at 142°E (line C on the map in Fig. 1 in the main text).

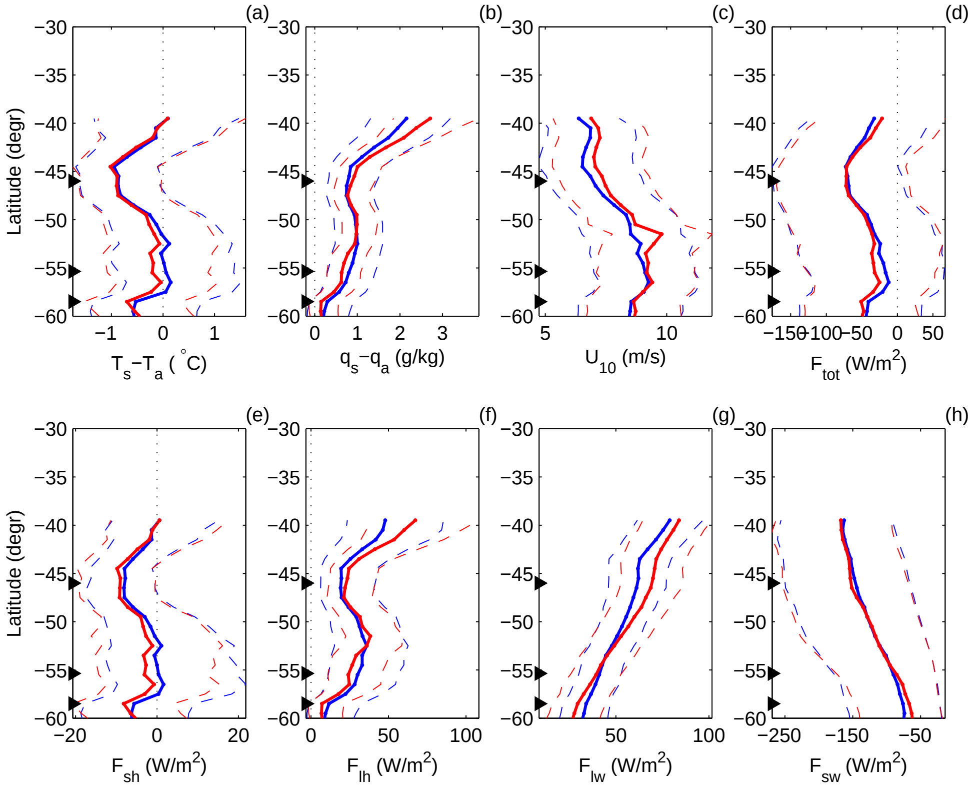

Supplementary Figure 10: As in Supplementary Fig. 7, but for a profile at 60°W (line E on the map in Fig. 1 in the main text).

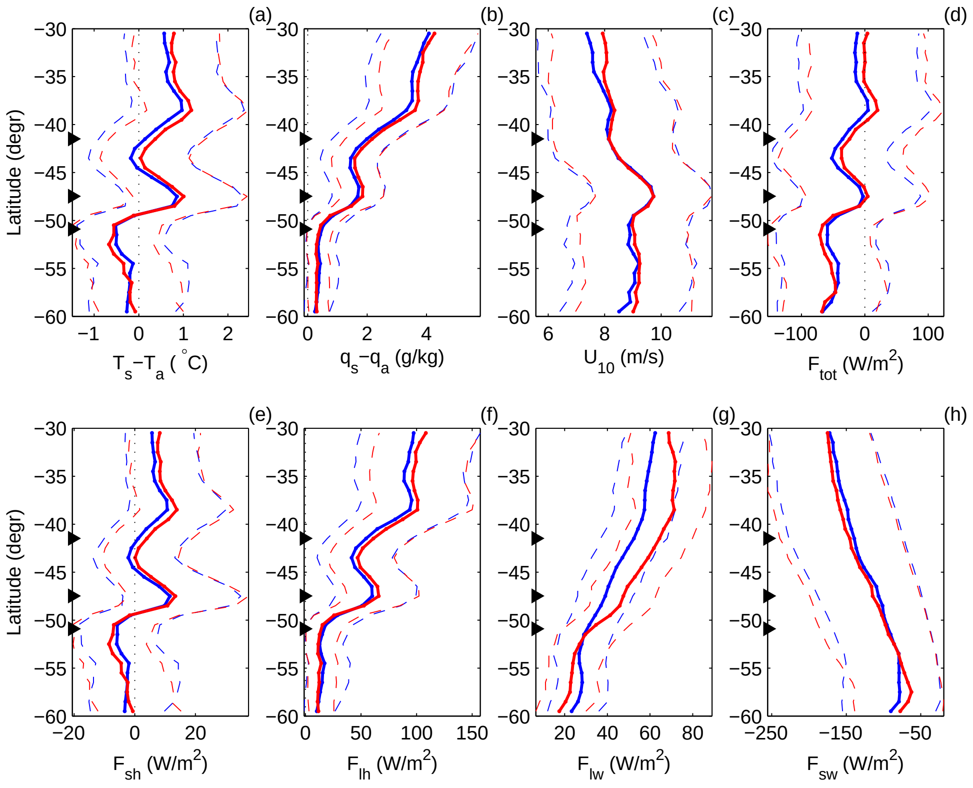

Supplementary Figure 11: As in Supplementary Fig. 7, but for a profile at 45°W (line F on the map in Fig. 1 in the main text).

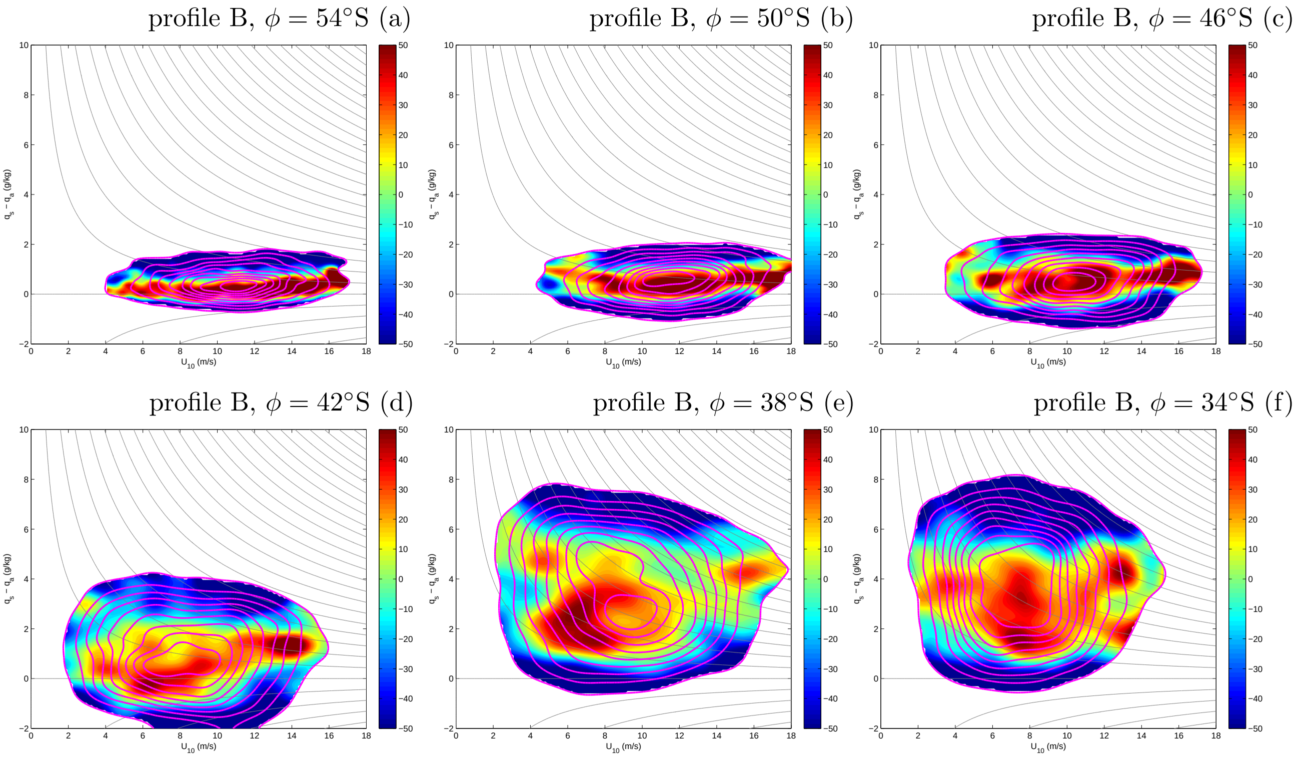

Supplementary Figure 12: Joint pdfs of the wind speed U_{10} and the humidity difference $q_s - q_a$ in points along the profile at 48°E (line B on the map in Fig. 1) between 54°S and 34°S (a–f), equally spaced at 4°-intervals. Magenta lines show the pdfs in the 1985–1994 decade, with contours drawn from 0.1 to 0.9, every 0.1 (relative values). The colors show the relative change (in %) of the pdfs during the analysis period. Thin gray lines show contours of constant latent heat flux (drawn every 30 W/m^2).

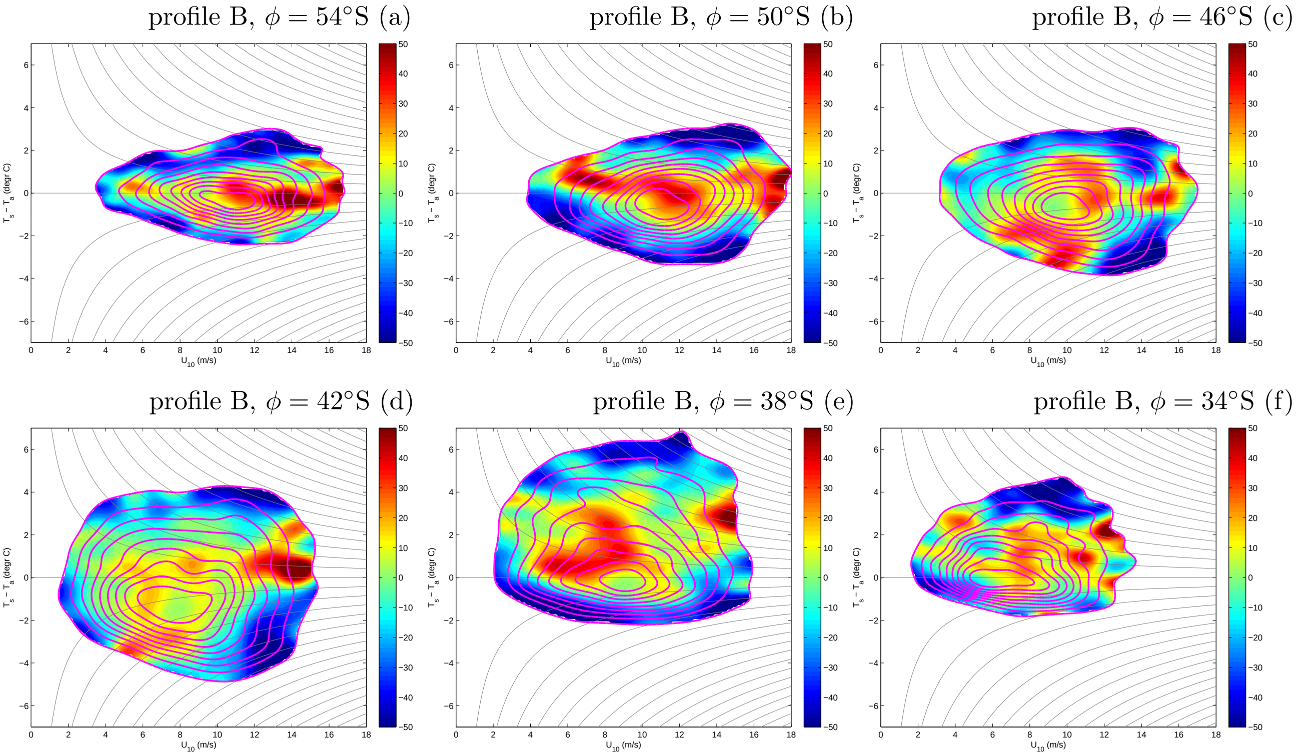

Supplementary Figure 13: As in Fig. 12, but for the joint pdfs of U_{10} and the temperature difference $T_s - T_a$. Thin gray lines show contours of constant sensible heat flux (drawn every 10 W/m^2).

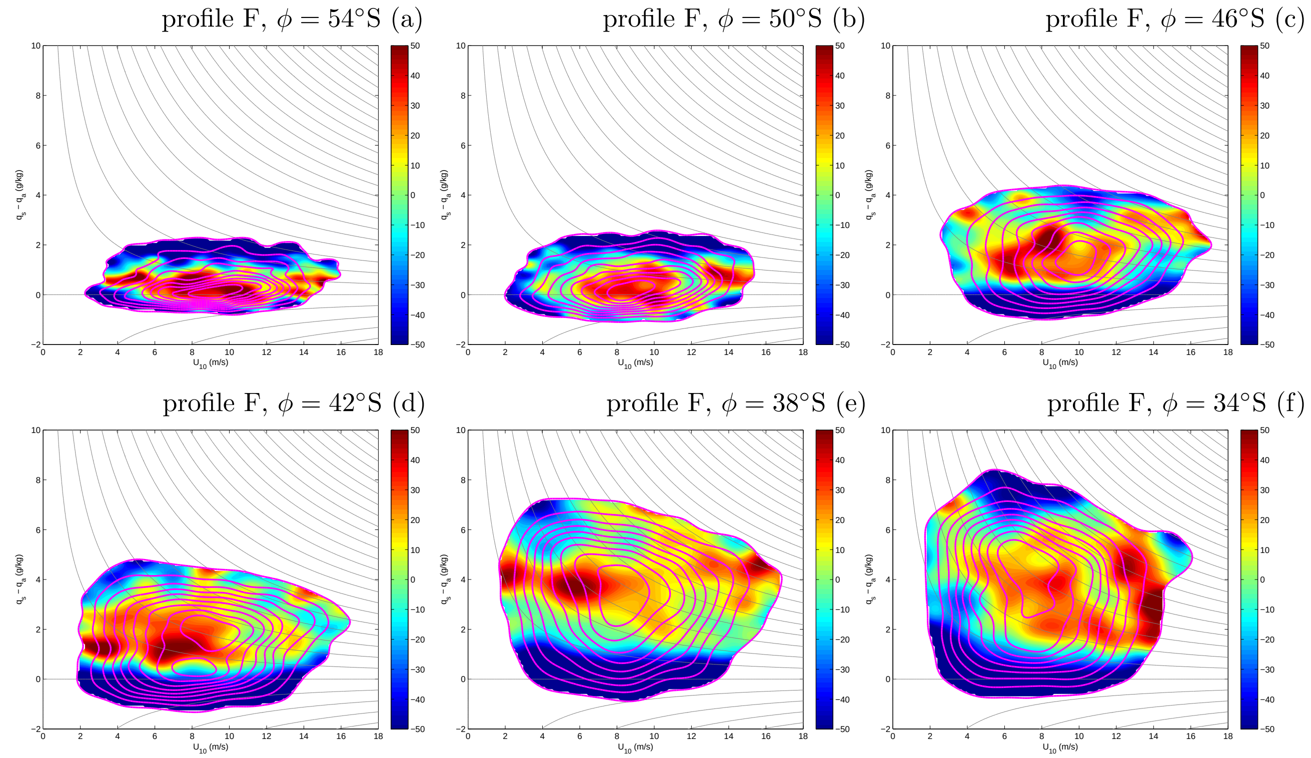

Supplementary Figure 14: As in Fig. 12, but for the profile at 315°E (line F on the map in Fig. 1).

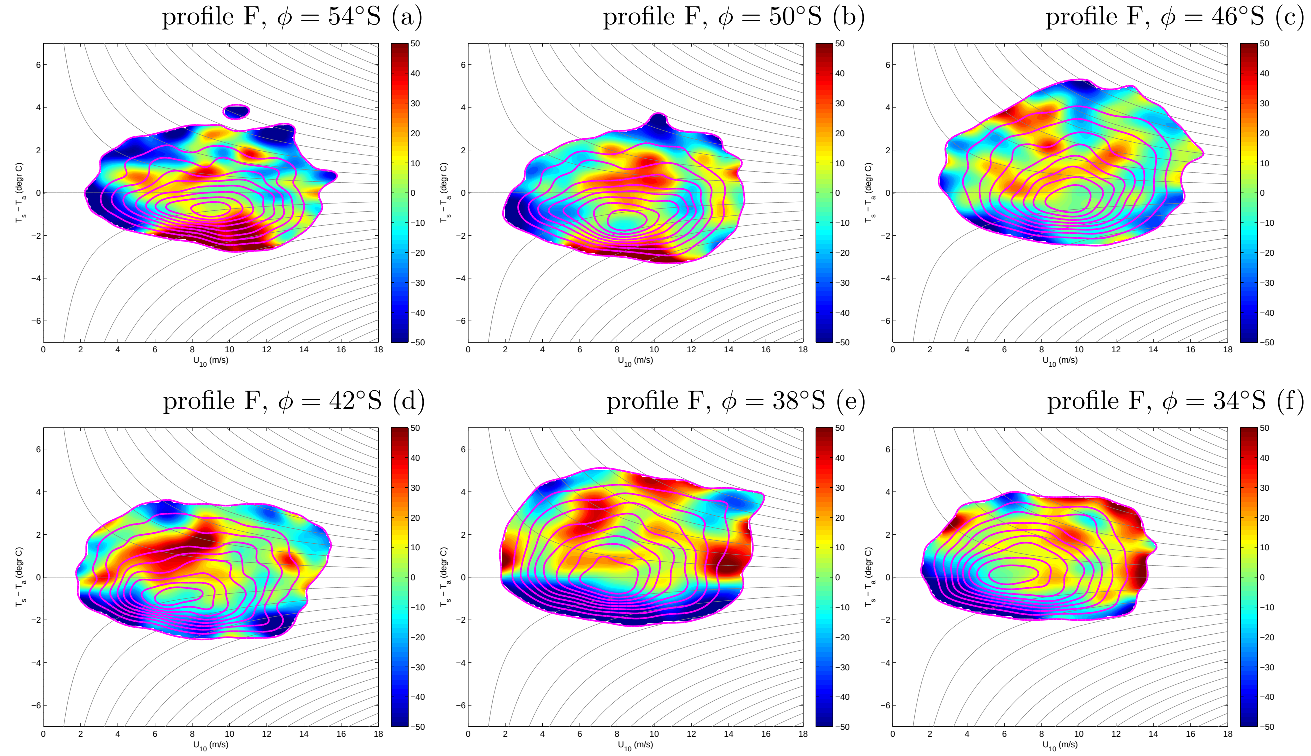

Supplementary Figure 15: As in Fig. 13, but for the profile at 315°E (line F on the map in Fig. 1).

Supplementary Note S2

Detection of periodicity in the analyzed spectrograms

In terms of the dependence of the power spectral density P on frequency f, the spectrograms analyzed in this work can be divided into two groups: power law, with $P(f) \sim f^{-\alpha}$, $\alpha > 1$ (for $\alpha = 2$ we speak of a red-noise spectrum); and exponential, with $P(f) \sim \exp(-\alpha f)$. Spectra of T_s, F_{sw} and F_{lw} belong to the first group, spectra of F_{lh}, F_{sh}, T_a, U_{10} and q_a – to the second group (Fig. 7 in the main paper and Supplementary Figs. 16, 18).

For white noise spectra, i.e., those with frequency-independent power density, an established, exact method exists for testing the significance of periodic variations against the background noise – the Fisher's g-statistic (see, e.g., [14]). However, no analogous exact method exists for non-white noise spectra. Established tests, based, e.g., on smoothing of the periodogram, are biased, because they have a strongly non-Gaussian distribution which formally makes them inappropriate for standard least-squares goodness-of-fit test.

In this work, two "measures of importance" of the peaks associated with the BAM signal are used, as detailed below. When interpreting the results, it should be kept in mind that the final spectrograms are averages of 513 spectrograms estimated from the subsets of data; additionally, they have been smoothed after averaging. This procedure, possible thanks to the sufficient length of the analyzed time series, tends to smooth out spurious peaks and leave only the robust ones, repeatedly occurring in the majority of the individual spectrograms. The above-mentioned tools used in the analysis are:

1. The peak-enhancement factor, or the peak prominence λ_p, a characteristics similar to the prominence ratio used in acoustics (again, suitable only for approximately-white noise spectra), here defined as the ratio between the maximum power density within frequency range 0.0313–0.0391 day^{-1} (BAM) to the minimum power density within frequency range 0.0166–0.0293 day^{-1}. For spectra with power density decreasing with f – including power-law and exponential spectra analyzed here – it is expected that $\lambda_p < 1$. The larger λ_p, the higher the peak (or, more precisely, its "left" slope in the spectral plot).
2. The height of the BAM-peak above the upper 95% prediction bounds associated with an exponential/power law fit to the spectrogram. (It is important not to confuse the prediction bounds for new observations with the confidence bounds for the coefficients of the fitted function. The prediction bounds measure the confidence that the new observation lies within the interval given a single predictor value – in this case, the BAM frequency.) The procedure for each analyzed spectrogram was as follows:

   - Remove from the spectrogram data points corresponding to the BAM frequency range in order to obtain the fit independent of these data points.
   - Fit an $a\exp(-\alpha f)$ or af^{α} function to the spectrogram (depending on its shape), find the 95% confidence bounds on the fit coefficients and analyze the histogram of residuals to estimate the accuracy of the fit.
   - Find the 95% prediction bounds for new observations for the whole frequency range.
   - Check whether the spectrogram data within the BAM frequency range lies above or below the upper prediction bound.

   The example results for the zonally-averaged spectrograms of F_{lh} (those shown in Fig. 7a in the main text) are shown in Supplementary Fig. 16.

Once more, neither of the two methods provides a final, conclusive answer regarding the significance of the BAM-peak. Moreover, the second methods seems too strict in situations when the BAM-peak is the global maximum of the spectrum, but does not exceed the prediction bounds – like in the example shown in Supplementary Fig. 16c. Nevertheless, our attention should be directed to regions where both methods suggest the existence of the BAM signal.

An additional indication of the robustness of the results provide the NCEP-DOE data, from which a very similar spatial pattern of λ_p is obtained, as shown in Supplementary Fig. 17. The spectrograms for zonally-averaged NCEP-DOE data are also very similar to those obtained from the OAFlux data (not shown).

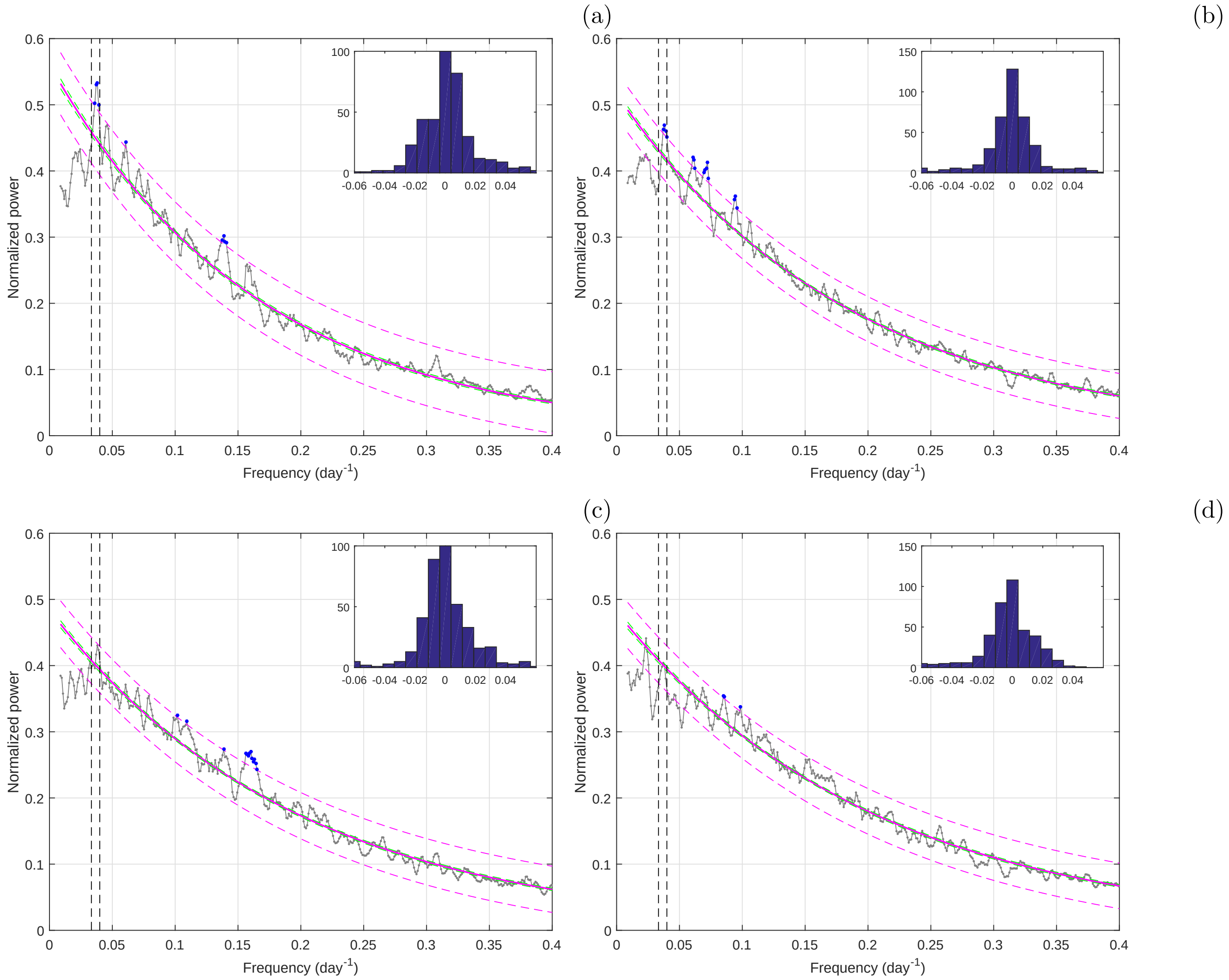

Supplementary Figure 16: Examples of normalized power spectra of zonally-averaged F_{lh} (a,b) and F_{sh} (c,d) data at two selected latitudes: 35°S (a,c) and 40°S (b,d) – see also Fig. 7 in the main text. Dashed vertical lines mark the range of BAM-frequencies. Continuous magenta lines show the exponential fit to the data, dashed green lines – 95% confidence bounds on the fitted curve, dashed magenta lines – 95% prediction bounds on new observations. Points above the upper prediction bounds are marked in blue. The insets show the histograms of the residuals of the fits.

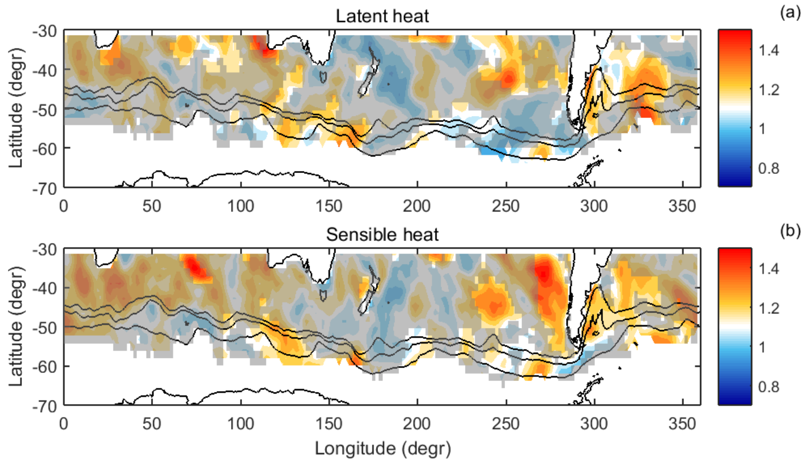

Supplementary Figure 17: Maps of the peak-enhancement factor λ_p for the spectra of F_{lh} (a) and F_{sh} (b) obtained from the NCEP-DOE data, analogous to those shown in Fig. 8 in the main text.

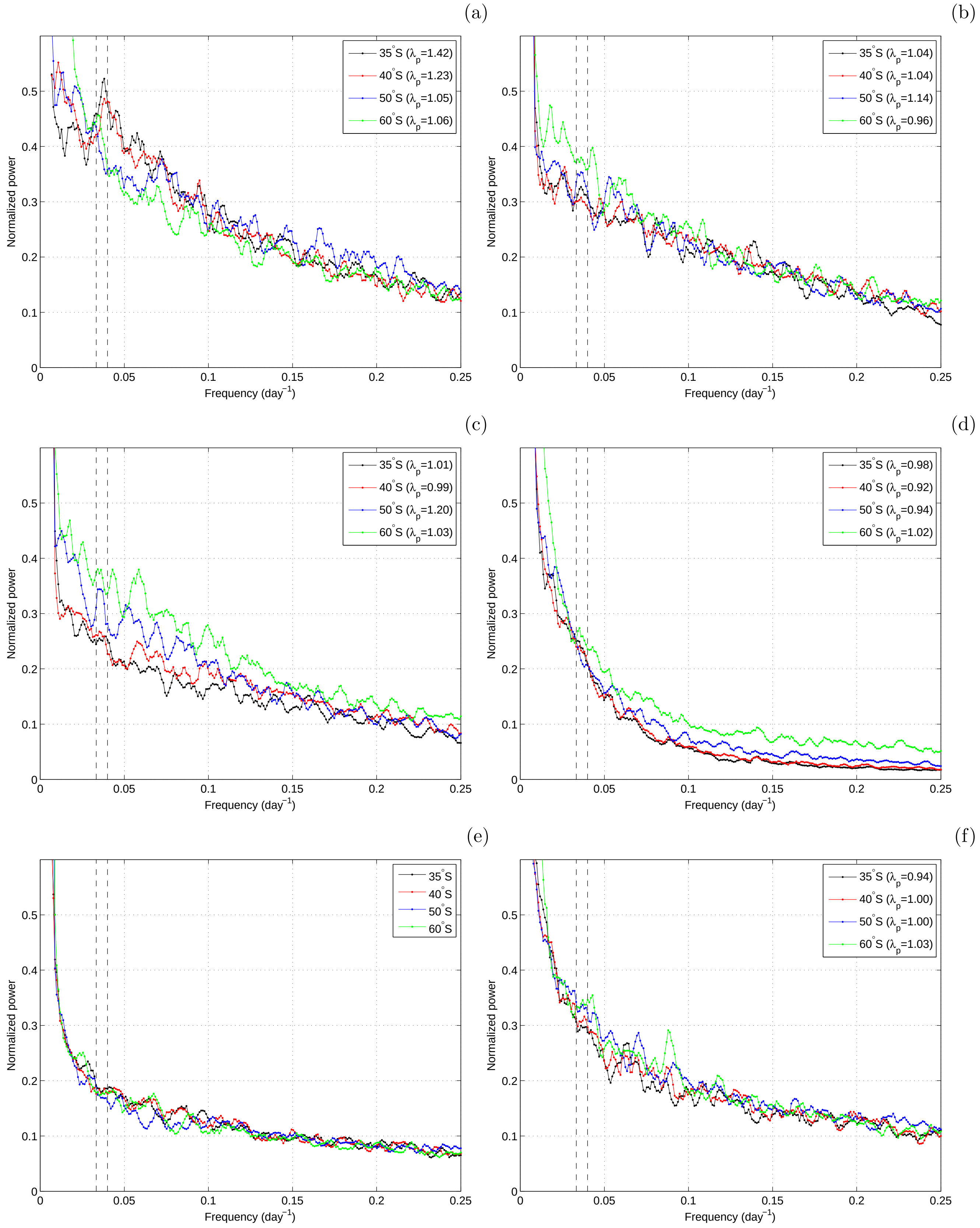

Supplementary Figure 18: Normalized power spectra of zonally-averaged data at four selected latitudes for U_{10} (a), q_a (b), T_a (c), T_s (d), F_{sw} (e), and F_{lw} (f). Dashed vertical lines mark the range of frequencies corresponding to periods of 25–30 days. See Fig. 7 in the main text for corresponding spectra of F_{lh} and F_{sh}.

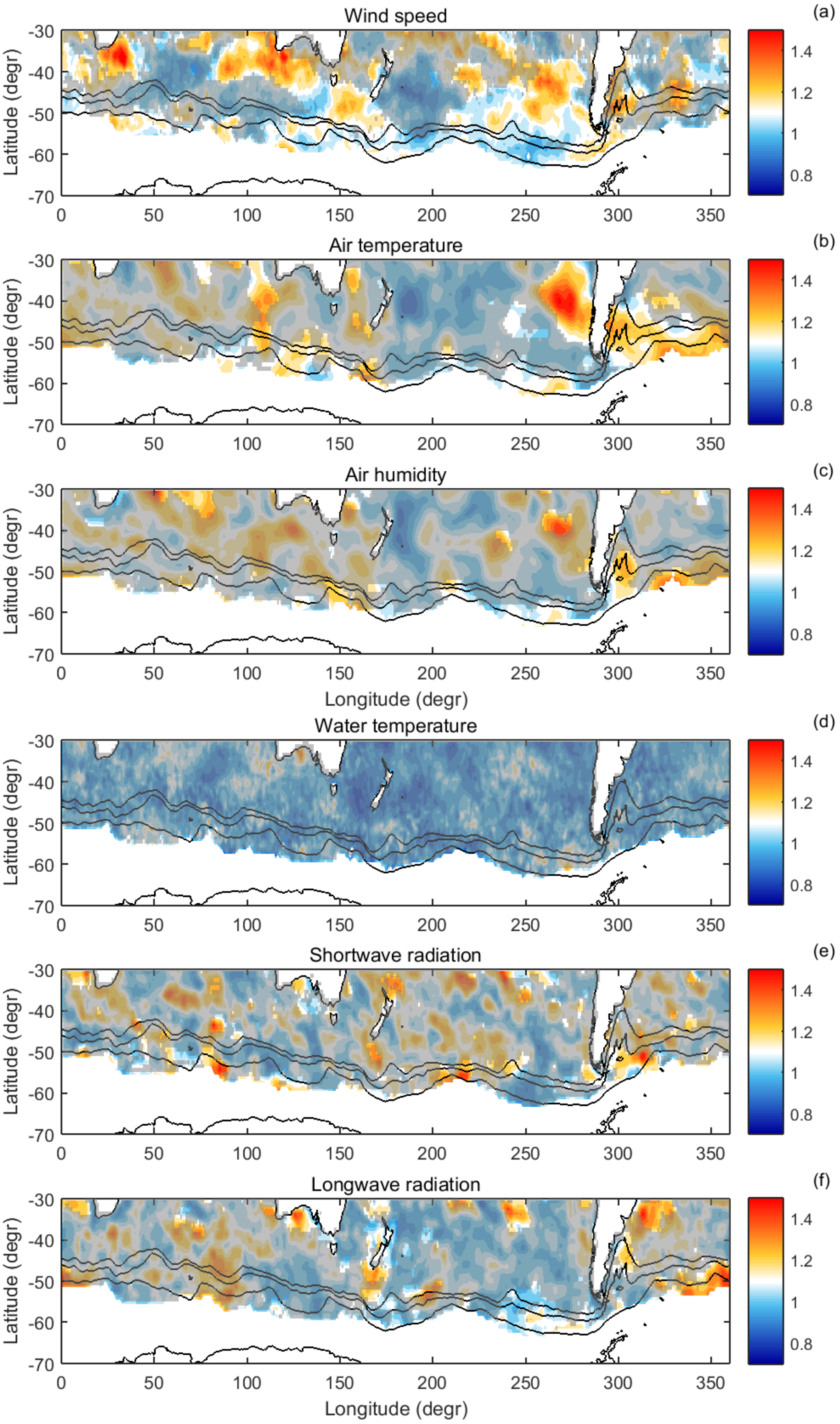

Supplementary Figure 19: Maps of the peak-enhancement factor λ_p for the spectra of U_{10} (a), T_a (b), q_a (c), T_s (d), F_{sw} (e), and F_{lw} (f). See Fig. 8 in the main text for corresponding maps of λ_p for F_{lh} and F_{sh}.

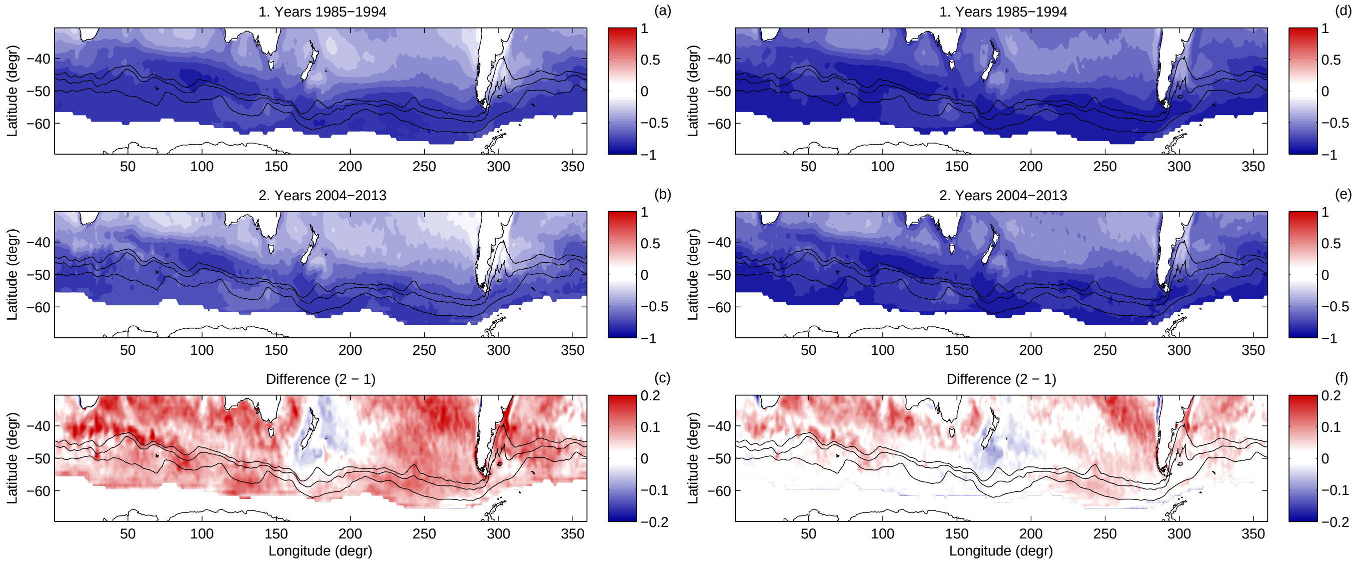

Supplementary Figure 20: Correlation coefficient of F_{lh} (a–c) and F_{sh} (d–f) with the air temperature T_a in the first (a,d) and last (b,e) decade of the analyzed period, and the change between them (c,f).

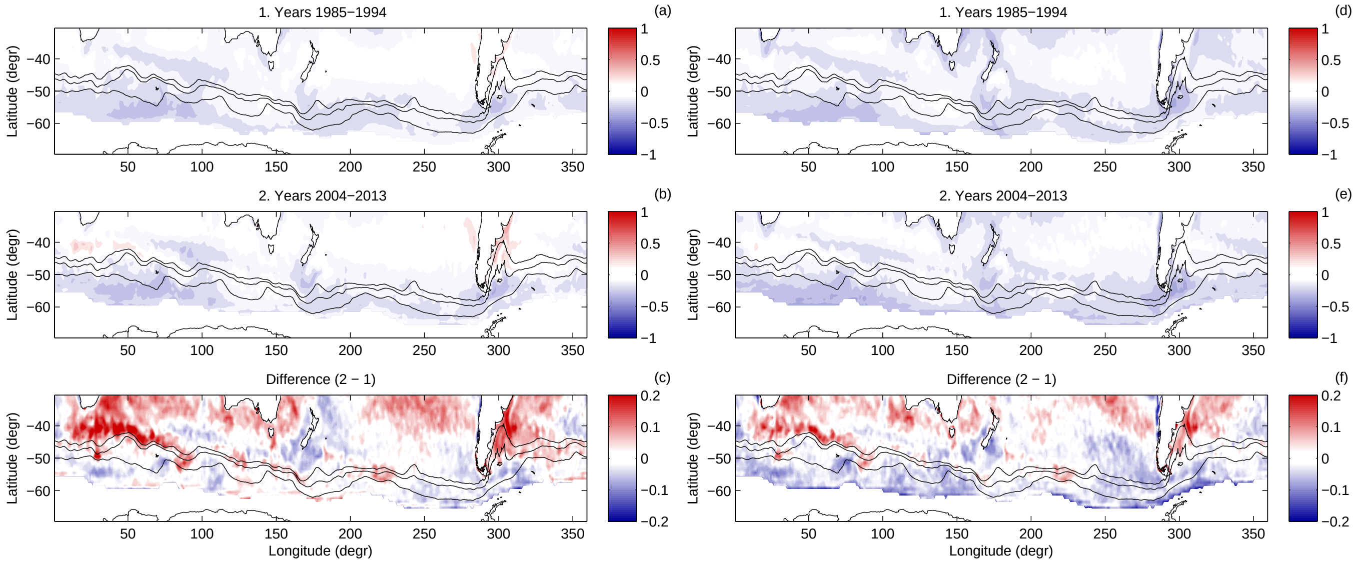

Supplementary Figure 21: As in Supplementary Fig. 20, but for the correlation coefficient of F_{lh} and F_{sh} with the sea surface temperature T_s.

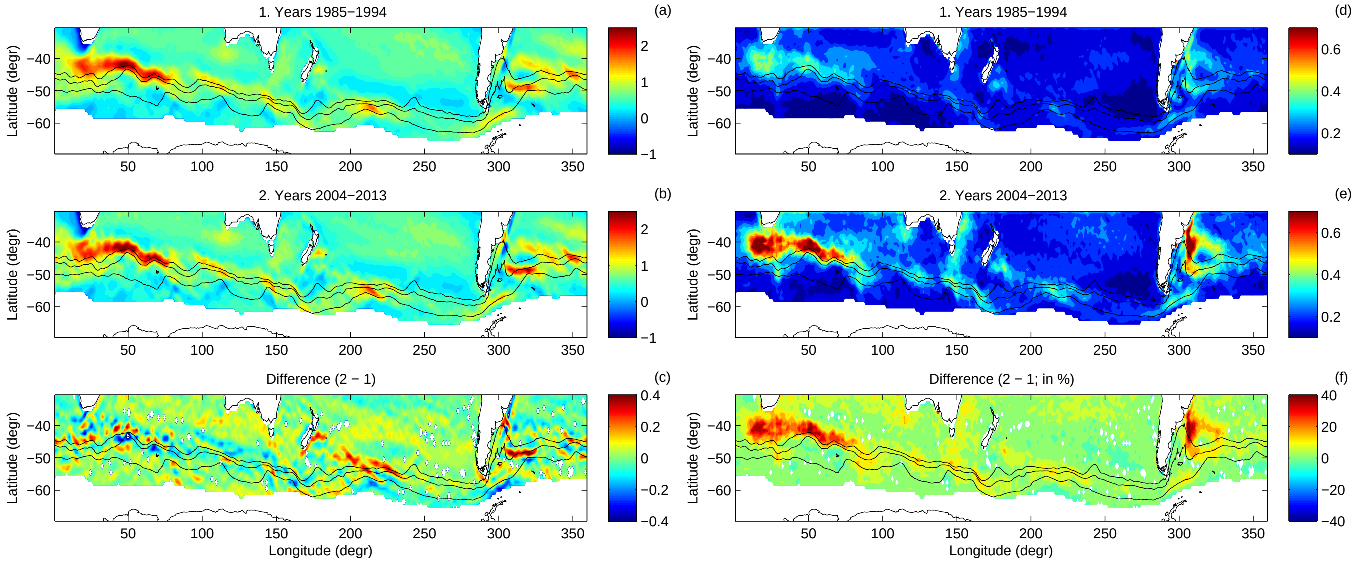

Supplementary Figure 22: As in Supplementary Fig. 20, but for the median (a–c) and standard deviation (d–f) of the meridional gradient of the sea surface temperature, $dT_s/d\phi$ (in degr C/degr lat).

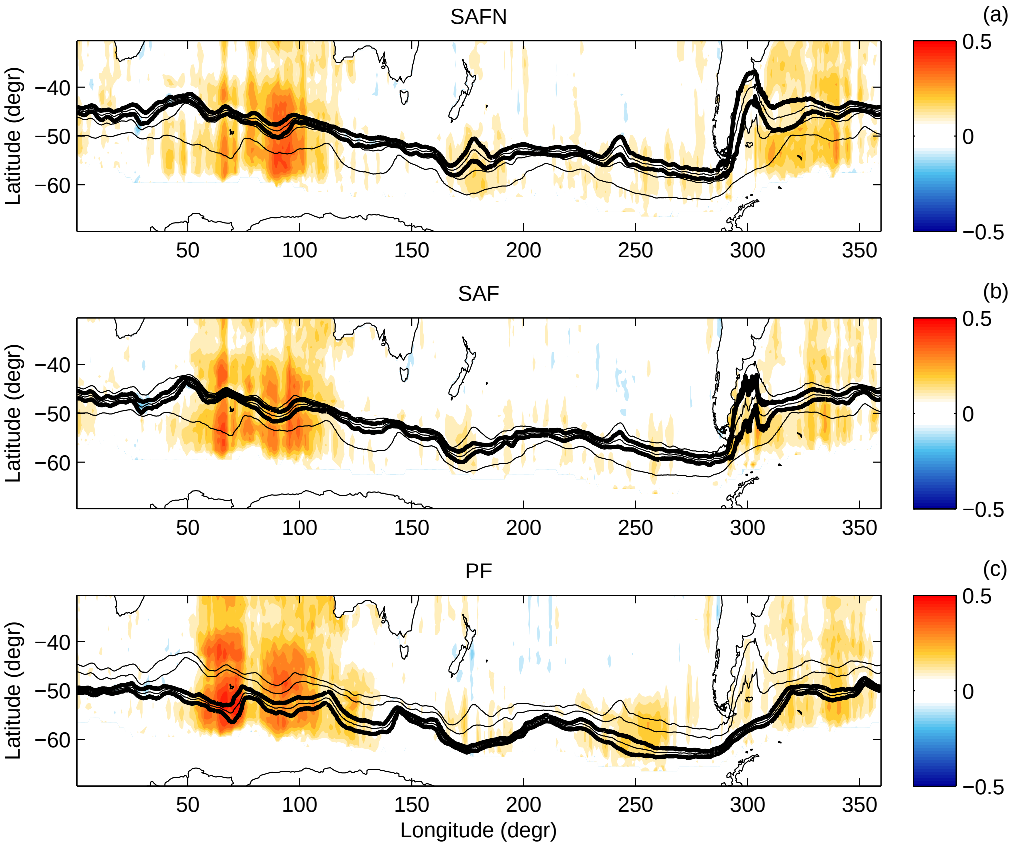

Supplementary Figure 23: Maps of the correlation coefficient between F_{sh} and the position of the three main ACC fronts: SAFN (a), SAF (b) and PF (c). See Methods for details, and Fig. 9 in the main text for corresponding maps for F_{lh}.

(a)

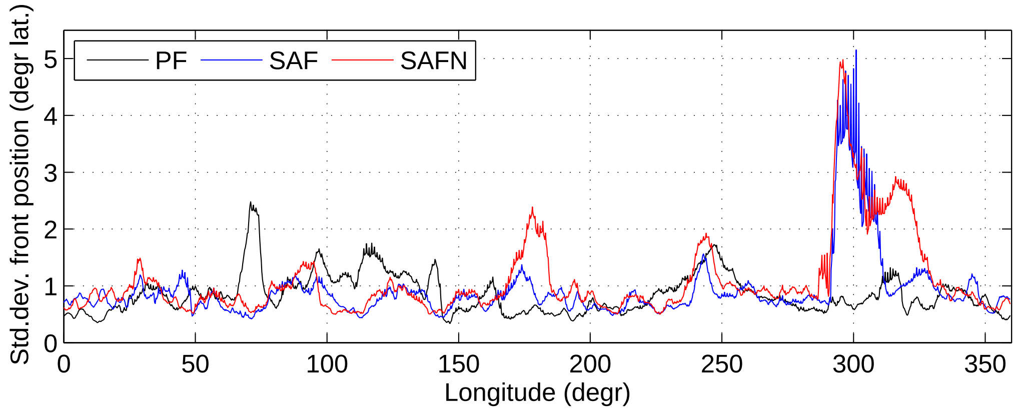

(b)

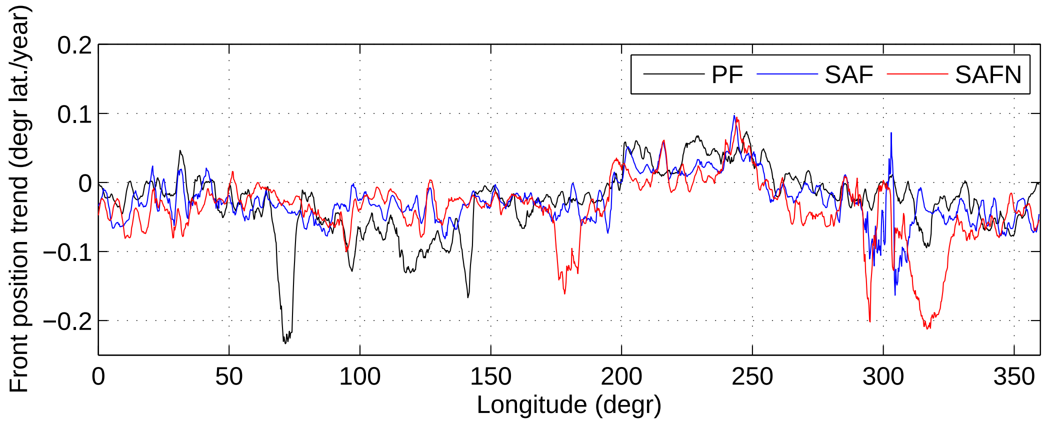

Supplementary Figure 24: Standard deviation (in degr. lat.; a) and linear trend (degr. lat/year; b) of the latitude position of the three major ACC fronts in the period 1992-2013. Negative values in (b) correspond to a southward shift of the fronts.

References

[1] L. Yu and R.A. Weller. Objectively analyzed air–sea heat fluxes for the global ice-free oceans (1981–2005). *Bull. American Meteorol. Soc.*, 88:527–539, 2007.

[2] S.H. Chou, E. Nelkin, J. Ardizzone, and R.M. Atlas. A comparison of latent heat fluxes over global oceans for four flux products. *J. Climate*, 17:3973–3989, 2004.

[3] L. Feng and J. Li. A comparison of latent heat fluxes over global oceans for ERA and NCEP with GSSTF2. *Geophys. Res. Lett.*, 33:L03810, 2006.

[4] L. Yu, X. Jin, and R.A. Weller. Multidecade global flux datasets from the Objectively Analyzed air-sea Fluxes (OAFlux) project: latent and sensible heat fluxes, ocean evaporation, and related surface meteorological variables. Technical report, Woods Hole Oceanographic Institution, OA-2008-01, 2008. 64 pp.

[5] A. Santorelli, R.T. Pinker, A. Bentamy, K.B. Katsaros, W.M. Drennan, A.M. Mestas-Nuñez, and J.A. Carton. Differences between two estimates of air-sea turbulent heat fluxes over the Atlantic Ocean. *J. Geophys. Res.*, 116:C09028, 2011.

[6] S.R. Smith, P.J. Hughes, and M.A. Bourassa. A comparison of nine monthly air-sea flux products. *Int. J. Climatology*, 31:1002–1027, 2011.

[7] L. Yu, Z. Zhang, S. Zhong, M. Zhou, Z. Gao, H. Wu, and B. Sun. An inter-comparison of six latent and sensible heat flux products over the Southern Ocean. *Polar Res.*, 30:10167, 2011.

[8] V. Faure, M. Arhan, S. Speich, and S. Gladyshev. Heat budget of the surface mixed layer south of Africa. *Ocean Dyn.*, 61:1441–1458, 2011.

[9] S. Gao, L.S. Chiu, and C.-L. Shie. Trends and variations of ocean surface latent heat flux: Results from GSSTF2c data set. *Geophys. Res. Lett.*, 40:1–6, 2013.

[10] M. Kanamitsu, W. Ebisuzaki, J. Woollen, S.-K. Yang, J.J. Hnilo, M. Fiorino, and G.L. Potter. NCEPDOE AMIP-II Reanalysis (R-2). *Bull. Amer. Meteor. Soc.*, 83:1631–1643, 2002.

[11] C.-L. Shie, K. Hilburn, L.S. Chiu, R. Adler, I.-I. Lin, E. Nelkin, J. Ardizzone, and S. Gao. The Goddard Satellite-based Surface Turbulent Fluxes dataset – Version 3 (GSSTF3). Distributed via Goddard Earth Sciences (GES) Data and Information Services Center (DISC), 2012. doi:10.5067/MEASURES/GSSTF/DATA301.

[12] K. Fennig, A. Andersson, S. Bakan, C. Klepp, and M. Schroeder. Hamburg Ocean Atmosphere Parameters and fluxes from Satellite data – HOAPS 3.2 – monthly means/6-hourly composites. Satellite Application Facility on Climate Monitoring, 2012. doi:10.5676/EUM_SAF_CM/HOAPS/V001.

[13] A. Andersson, K. Fennig, C. Klepp, S. Bakan, H. Grassl, and J. Schulz. The Hamburg Ocean Atmosphere Parameters and fluxes from Satellite data – HOAPS-3. *Earth Syst. Sci. Data*, 2:215–234, 2010.

[14] D.B. Percival and A.T. Walden. *Spectral Analysis for Physical Applications.* Cambridge, UK: Cambridge University Press, 491 pp., 1993.
